# Identification of programmed cell death-related subtypes reveals immune heterogeneity and therapeutic divergence in colon cancer

**DOI:** 10.7150/thno.126314

**Published:** 2026-02-18

**Authors:** Peng Xia, Ying Qu, Quanzhong Liu, Mengyan Zhu, Bin Huang, Wei Wu, Kening Li, Lingxiang Wu, Ruohan Zhang, Yingli Lv, Qianghu Wang

**Affiliations:** 1 School of Biological Science & Medical Engineering, Southeast University, Nanjing 211189, China.; 2 Department of Bioinformatics, Nanjing Medical University, Nanjing 211166, China.; 3 Institute for Brain Tumors, Jiangsu Collaborative Innovation Center for Cancer Personalized Medicine, Nanjing Medical University, Nanjing 211166, China.; 4 College of Bioinformatics Science and Technology, Harbin Medical University, Harbin 150081, China.; 5 Department of Neurosurgery, Beijing Tiantan Hospital, Capital Medical University, Beijing 100070, China.; 6 The Affiliated Cancer Hospital of Nanjing Medical University, Jiangsu Cancer Hospital, Jiangsu Key Laboratory of Innovative Cancer Diagnosis & Therapeutics, Nanjing 210009, China.; 7 Department of Pathology, Jiangsu Province Hospital and the First Affiliated Hospital of Nanjing Medical University, Nanjing 210029, China.

**Keywords:** colon adenocarcinoma, molecular subtypes, therapy resistance, tumor microenvironment

## Abstract

**Rationale:**

Therapy resistance remains a critical challenge in colon adenocarcinoma (COAD). The dysregulation of programmed cell death (PCD) pathways significantly influences therapeutic response, but its integrated role in shaping the tumor microenvironment (TME) and driving clinical heterogeneity in COAD is poorly defined.

**Methods:**

We established a Programmed Cell Death-related Subtype (PCDS) classification by integrating 12 PCD pathways across transcriptomic data from 1,140 COAD patients using non-negative matrix factorization (NMF). The subtypes were validated in independent RNA-sequencing cohorts. We characterized the genomic, TME, and therapeutic features of each PCDS using multi-omics data analysis, and computational drug repositioning. Molecular docking and in silico drug sensitivity analyses were employed to evaluate candidate drugs.

**Results:**

We identified three robust subtypes, including PCDS1 (immune-activated), PCDS2 (WNT and TP53 signaling activation), and PCDS3 (mesenchymal and T-cell dysfunction/exclusion). PCDS3, enriched with inflammatory cancer-associated fibroblasts (iCAFs), exhibited the poorest prognosis and dual resistance to both chemotherapy and immunotherapy (>80% non-response). Analysis of single-cell and spatial transcriptomics data revealed the activation of MDK-SDC2 ligand-receptor axis between tumor cells and fibroblasts in PCDS3, spatially associated with T-cell dysfunction and exclusion. Computational drug repositioning identified the sunitinib as having selective potency against PCDS3 tumors, showing significantly lower IC50 values and high-affinity binding to SDC2 in molecular docking.

**Conclusions:**

This study defines a novel molecular subtype for COAD, linking PCD dysregulation to distinct TME remodeling and therapeutic outcomes. Targeting the MDK-SDC2 axis with agents such as sunitinib may offer a promising strategy to overcome stromal-mediated immunotherapy resistance in the most lethal PCDS3 tumors.

## Introduction

Colon adenocarcinoma (COAD) is the third most commonly diagnosed cancer and the second leading cause of cancer-related deaths, with an estimated 903,859 deaths annually 1. Despite advances in multiple treatment strategies, a substantial proportion of patients develop therapeutic resistance, particularly those with metastatic disease, where 5-year survival rates plummet below 15% 2-5. This clinical crisis stems largely from the molecular heterogeneity of COAD, which drives different treatment responses and promotes both intrinsic and acquired resistance mechanisms 5-10. Therapeutic outcomes in COAD have not yet attained the desired level of efficacy, underscoring the critical need for discovering innovative classification strategies to refine risk stratification and guide clinical interventions.

Widely adopted stratification frameworks, such as the Consensus Molecular Subtypes (CMS), have highlighted the biological diversity of COAD 11. Becht et al. integrated the tumor microenvironment with the CMS classification of colorectal cancer and reported that colorectal cancer molecular subtypes are closely associated with specific microenvironmental signatures 12. The favorable-prognosis microsatellite instability (MSI)-enriched CMS1 subtype is marked by the overexpression of genes associated with cytotoxic lymphocytes, while the poor-prognosis mesenchymal subtype (CMS4) shows expression of both lymphocyte and monocytic cell marker. In contrast, the canonical (CMS2) and metabolic (CMS3) subtypes, both associated with intermediate prognosis, are characterized by low immune and inflammatory signatures. Nevertheless, CMS classification does not fully capture the complexity of programmed cell death (PCD) pathways dysregulation, which are key regulators of therapeutic response. PCD pathways (e.g., apoptosis, necroptosis, pyroptosis, ferroptosis) constitute critical determinants of drug sensitivity, their integrated alterations and clinical implications in COAD remain poorly defined 13-17. Critically, no existing subtype system predicts vulnerabilities in therapeutically recalcitrant populations by integrating PCD networks with tumor microenvironment (TME).

The TME of therapy-resistant COAD-marked by stromal remodeling and immune evasion-remains poorly understood through bulk transcriptomic profiling 18, 19. Single-cell RNA sequencing (scRNA-seq) offers unprecedented resolution to analyze cellular diversity within these recalcitrant niches. ScRNA-seq technology provides high resolution insights into heterogeneity of TME and has revealed key features of resistance, including cancer-associated fibroblast (CAF) plasticity and T-cell dysfunction 20-23. Critically, aberrant intercellular communication between malignant cells and stromal compartments orchestrates some signaling pathways that promote therapy resistance 24-26, offering new opportunities for therapeutic intervention.

**Figure 1:**
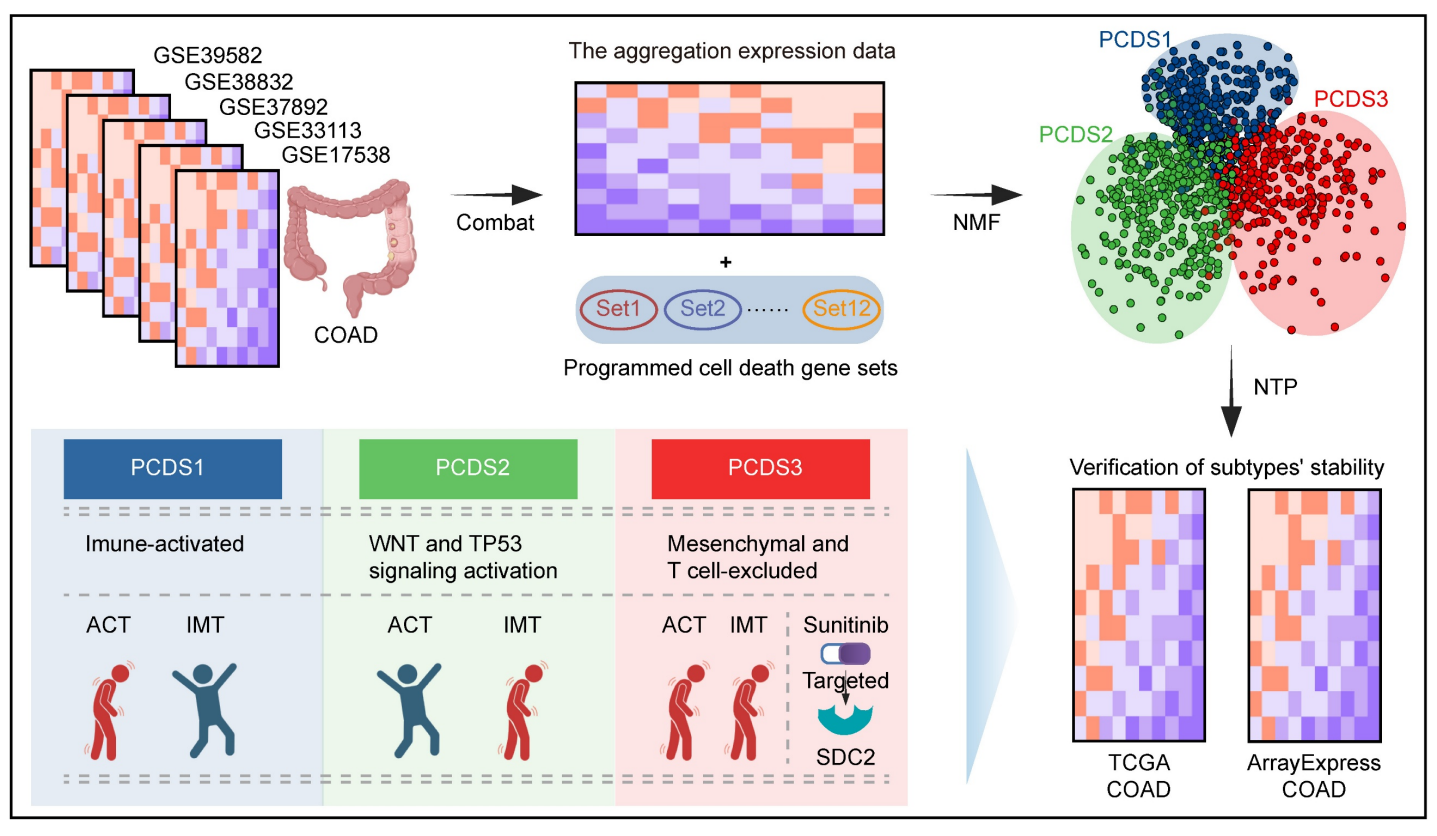
** Schematic of the study design.** COAD: colon adenocarcinoma; NMF: Non-negative Matrix Factorization; PCDS: Programmed Cell Death-related Subtype; NTP: Nearest Template Prediction; ACT: Adjuvant chemotherapy; IMT: Immunotherapy.

Here, we establish Programmed Cell Death-related Subtype (PCDS) to redefine treatment-resistant COAD (**Figure 1**). By integrating 12 PCD pathways across 1,140 patients, we identify three different molecular subtypes: PCDS1 (immune-activated), PCDS2 (WNT and TP53 signaling activation), and PCDS3 (mesenchymal and T-cell dysfunction and exclusion). Among these, PCDS3 exhibits the poorest prognosis and shows resistance to both chemotherapy and immunotherapy. It's characterized by epithelial-mesenchymal transition (EMT) and inflammatory pathways, enrichment of iCAFs, and spatial MDK-SDC2 interactions that correlate with T cell exclusion. Computational drug repositioning further identified the multi-kinase inhibitor sunitinib as having selective efficacy against this lethal subtype, which was supported from both in silico binding affinity and drug sensitivity analyzing. This work combined PCD, TME remodeling, and therapeutic discovery, providing a selectable framework for targeting the most lethal COAD subtype.

## Materials and Methods

### Patients and tumor samples

We collected five COAD microarray datasets (GSE17538 27, 28, GSE33113 29, GSE39582 30, GSE38832 31, and GSE37892 32) encompassing 1,140 patients from the GEO database (**Table 1**). All datasets were generated using the “Affymetrix Human Genome U133 Plus 2.0 Array” platform (GPL570). Among these five datasets, GSE17538, GSE33113, and GSE39582 contain relapse-free survival (RFS) data. The RNA expression profiles, gene mutation data, and corresponding clinical information for colon adenocarcinoma (COAD) were acquired from The Cancer Genome Atlas (TCGA) database (https://xenabrowser.net/datapages/). The neoantigen counts of both SNV- and Indel-derived neoantigens for the TCGA COAD cohort were obtained from the supplemental data of Thorsson et al. 33. A large-scale COAD RNA expression dataset (accession number: E-MTAB-12862) 34 was obtained from ArrayExpress. An additional dataset (GSE235919) 35, which contains RNA-seq data from 34 patients with metastatic colorectal cancer treated with immune checkpoint blockade (ICB), was used as supplementary evidence for evaluating ICB treatment response. For single-cell sequencing analysis, dataset GSE200997 36 was selected owing to its inclusion of matched bulk RNA expression profiles, enabling directly integrated molecular subtyping. Two additional single-cell datasets, GSE178341 37 and GSE236581 38, were also incorporated for relevant validation. The processed spatial transcriptome (ST) data from the primary lesions of four colorectal cancer patients were downloaded from http://www.cancerdiversity.asia/scCRLM/ 39.

### Bulk RNA data pre-processing

First, we normalized each microarray dataset using the Frozen Robust Multi-array Analysis (fRMA) algorithm 40. Probeset identifiers were mapped to gene symbols with the R package “hgu133plus2.db” (v3.2.3). For genes that correspond to multiple probesets, we used the average expression value of these probesets to represent the gene. Then, batch effects within five GEO datasets (GSE17538, GSE33113, GSE39582, GSE38832 and GSE37892) were removed using the ComBat 41 method in the R package “sva” (v3.34.0).

### Identifying the PCDS of COAD using NMF

To establish the PCDS classification framework, we extracted a curated list of 1,162 genes associated with 12 distinct PCD pathways from a published resource 42. These pathways encompass major PCD modalities with established roles in cancer biology and therapy response, including apoptosis, necroptosis, pyroptosis, ferroptosis, etc. Then, we performed non-negative matrix factorization (NMF) clustering on 1,140 COAD samples from the GEO database based the full 1,162 genes. Using the “NMF” R package (v0.27), we first executed preliminary NMF runs across a rank range of k = 3-7. The optimal number of clusters was determined as k = 3 based on maximal cophenetic correlation coefficient, followed by 30 replicate NMF iterations to ensure robust clustering. Samples were subsequently assigned to different clusters, and module-specific signature genes were identified using a three-step strategy: First, we extracted the basis matrix W (genes × modules) and coefficient matrix H (modules × samples). Second, an adaptive thresholding approach was applied whereby the 80% of all weights in W served as a global cutoff, and genes exceeding this threshold within a given module were designated as candidate signatures. Modules containing fewer than 10 qualifying genes retained their top 10 highest-weight genes. Third, to eliminate cross-module interference and ensure maximal subtype specificity, we retained only uniquely assigned genes by excluding those appearing in multiple modules. This stringent process yielded a final set of 303 mutually exclusive signature genes that robustly define the three PCDSs.

### Differential expression and gene functional enrichment analysis

The significantly overexpressed genes in each PCDS compared to others were identified using the “limma” 43 R package (v3.56.2). Genes with the criteria of an adjusted p value < 0.05 and fold change > 1.5 were defined as PCDS-specific up-regulated genes. These up-regulated genes were further used for gene functional enrichment analysis with the the Enrichr (https://maayanlab.cloud/Enrichr/), and the MSigDB hallmarks with p values < 0.05 were considered significant. Additionally, pathway activity scores were computed using the single sample gene set enrichment analysis (ssGSEA) from “GSVA” 44 R package (v1.48.3).

### Nearest template prediction of the PCDS

The R package “CMScaller” (v2.0.1) 45 was utilized for CMS analysis and Nearest Template Prediction (NTP). The 303 PCDS-specific marker genes, identified through NMF clustering, served as templates for the NTP algorithm. By applying the “ntp” function from CMScaller, we assigned robust PCDS labels with high reproducibility to COAD patients across both TCGA and ArrayExpress RNA-seq gene expression profiles, validated by bootstrap resampling (fdr < 0.05).

### Survival analysis

Using COAD cohorts from GEO, TCGA, and ArrayExpress, we comprehensively evaluated prognostic differences among three PCDSs. Survival analysis was conducted with the R package “survival” (v3.5.5), and Kaplan-Meier curves were generated using the ggsurvplot function from “survminer” R package (v0.4.9). Differential survival across PCDSs was assessed using the log-rank test. Furthermore, within each PCDS, we examined survival outcomes stratified by adjuvant chemotherapy (ACT) status to determine chemotherapy benefit. Survival analyses were performed under the unified RFS endpoint.

### Gene mutation analysis

The somatic mutation data of COAD patients called by Mutect software were downloaded from TCGA GDC website. The pipeline of the “maftools” 46 package (v2.6.5) was using to performe mutational signature analysis. Tumor mutation burden (TMB) was calculated using the “tmb” function.

### Immune infiltration and immune dysfunction and exclusion (TIDE) analysis

To evaluate immune cell infiltration levels across distinct PCDS patients, we employed the R package “IOBR” (v0.99.8) 47 to execute cibersort 48, thereby quantifying relative abundances of 22 immune cell types within each sample. The TIDE score can serve as a biomarker to predict response to immune checkpoint blockade, including anti-PD1 and anti-CTLA 49. We used the TIDE online tool (http://tide.dfci.harvard.edu/) to calculate T cell dysfunction and exclusion scores along with composite TIDE scores, enabling assessment of individual patients' response to immunotherapy.

### ScRNA-seq data analysis: unsupervised clustering and identification of cell subpopulations

We performed logarithmic normalization and linear regression using “NormalizeData” and “ScaleData” functions from “Seurat” 50 R package (v4.4.0). To remove batch effects across samples, all datasets were reintegrated via Canonical Correlation Analysis (CCA) prior to clustering. Subsequent analyses employed Seurat's pipeline: principal component analysis (“RunPCA”), neighborhood graph construction (“FindNeighbors”), and unsupervised clustering (“FindClusters”). For visualization, we reduced dimensionality using uniform manifold approximation and projection (UMAP) via “RunUMAP”, retaining the same principal components used for clustering. Cell identities were annotated based on established lineage-specific markers: B cells (*CD19*, *MS4A1*, *CD79B*, *CD79A*), Endothelial cells (*CLDN5*, *ENG*, *VWF*, *PECAM1*), Epithelial cells (*CD24*, *CEACAM5*, *EPCAM*, *KRT18*, *KRT8*), Fibroblasts (*ACTA2*, *DCN*, *COL3A1*, *COL1A1*), Mast cells (*KIT*, *CPA3*, *TPSAB1*), Myeloid cells (*FCGR3A*, *S100A8*, *CD14*, *CD68*, *LYZ*), NK cells (*GZMB*, *GNLY*, *PRF1*, *NKG7*), Plasma cells (*SDC1*, *TNFRSF17*, *MZB1*) and T cells (*CD3G*, *CD2*, *CD3E*, *CD3D*). Fibroblasts were isolated and subjected to subclustering analysis. Differentially expressed genes (DEGs) among fibroblast subclusters were identified using FindAllMarkers. Malignant tumor cells were predicted via the “copykat” 51 R package (v1.1.0).

### Construction of pseudobulk gene expression profile

To obtain sample-level gene expression profile from the scRNA-seq data, we constructed pseudobulk expression matrices by summing gene expression from all cells within each sample. Briefly, for each sample, gene expression values from all cells were summed in the normalized expression matrix, creating a sample-by-gene pseudobulk gene expression profile 52. The resulting pseudobulk gene expression profiles were used for downstream PCDS analysis, maintaining consistency with the bulk RNA-seq subtyping framework.

### Spatial transcriptome data analysis

We applied the standard Seurat workflow to analyze spatial transcriptomic data, specifically utilizing “SCTransform” 53 for normalization, “RunPCA” for dimensionality reduction, “FindNeighbors” and “FindClusters” for spatial domain identification, and “RunUMAP” for visualization. Subsequently, we employed the “SpaGene” 54 R package (v0.1.0) to detect colocalized ligand-receptor pairs and visualized these spatial interaction patterns using “SpatialDimPlot”.

### PCDS distribution of each cell type

We calculated the Ro/e for each cell type in different PCDS to quantify the PCDS preference of each cell type 55, 56. The expected cell numbers for each combination of cell types and PCDS were obtained from the chi-squared test. One cell type was identified as being enriched in a specific PCDS if Ro/e > 1. For most cell types, we used the Ro/e index (+++, Ro/e > 3; ++, 1 < Ro/e ≤ 3; +, 0.2 ≤ Ro/e ≤ 1; +/-, 0 < Ro/e < 0.2; and -, Ro/e = 0) 57 to define the cell type preference in a specific PCDS.

### Cell-cell interaction analysis

We used the “CellChat” 58 R package (v2.1.1) to infer cell-cell interactions between different fibroblast and tumor cell suclusters. The potential interaction intensity between two cell suclusters was predicted based on the expression of ligand-receptor pairs and the ligand-receptor interaction database. Ligand-receptor pairs with a p-value < 0.05 determined by CellChat were considered to be significant ligand-receptor interactions between different cell subsets. In addition, the “nichenetr” 59 R package (v2.2.0) was utilized to implement the NicheNet framework, enabling the assessment of functional activity for the inferred ligand-receptor interactions.

### Tissue microarray multiple immunofluorescence (mIF) assay

Tissue microarray slides containing 86 adjacent non-tumor tissues and 94 colorectal cancer tumor tissues were obtained from Shanghai Outdo Biotech Company. The mIF staining was performed using the following primary antibodies, including anti-Midkine antibody (Abcam, cat. no. ab52637), SDC2 monoclonal antibody (Proteintech, cat. no. 67088-1-Ig), anti-TIGIT antibody (Abcam, cat. no. ab243903), anti-pan Cytokeratin antibody (ZSGB-BIO, cat. no. ZM-0069), anti-FAP antibody (Abcam, cat. no. ab53066) and anti-CD3 antibody (Abcam, cat. no. ab16669). The nuclei were stained with 4,6-Diamidino-2-phenylindole (DAPI). All staining procedures were performed according to the manufacturers' protocols. Whole-slide images were acquired using a panoramic digital slide scanner (Pannoramic SCAN 150). Image visualization was performed using TissueFAXS Viewer. QuPath software (version 0.4.3) was used to quantify cells. Specifically, tumor and stromal regions were first delineated based on pan-cytokeratin (CK) expression, and individual cells were subsequently identified using DAPI nuclear staining. Single-positive, double-positive, and triple-positive cells for the markers of interest were identified according fluorescence intensity of specific marker, and cell counts were quantified for statistical analysis. The MDK-SDC2 co-localization ratio for each sample was defined as the proportion of MDK⁺FAP⁺SDC2⁺ tumor cells among all tumor cells. Samples were subsequently stratified into MDK-SDC2 co-localization-high and -low groups based on the median co-localization ratio across the tumor tissues. Similarly, the infiltration ratio of CD3⁺ T cells was calculated as the number of CD3⁺ T cells divided by the total number of DAPI⁺ nuclei. A cutoff value of 20% was applied to classify samples into high or low CD3⁺ T-cell infiltration groups for association analyses.

### Drug sensitivity prediction

Gene expression profiles and the half maximal inhibitory concentration (IC50) values of drugs from pharmacologically-treated cell lines were acquired from the Genomics of Drug Sensitivity in Cancer 2 (GDSC2) database (https://www.cancerrxgene.org/) 60, 61. We employed the “oncoPredict” 62 R package (v1.2) to investigate gene expression-drug sensitivity relationships, specifically applying the calcPhenotype function to compute drug sensitivity profiles for COAD patients across both TCGA and ArrayExpress RNA-seq datasets. Subsequently, we calculated correlation coefficients between gene expression and IC50 values for each compound, drugs that showed a negative correlation with the expression of SDC2 and had a p value < 0.05 were considered potential targeted drugs for SDC2.

### Molecular docking by the molecular operating environment (MOE)

We retrieved the chemical structure of sunitinib in SDF format from the PubChem database (https://pubchem.ncbi.nlm.nih.gov/) and acquired the three-dimensional protein structure of SDC2 from the Protein Data Bank (PDB, https://www.rcsb.org/), subsequently performing molecular docking between the protein and small molecule drug using MOE software (v2019.0101), which ranked ligand-receptor binding conformations based on energy scores (S-score).

### Quantification and statistical analysis

We used the Wilcoxon rank-sum test or one-way ANOVA for group comparisons, and applied the Kaplan-Meier method to analyze RFS across different risk groups. All analyses were conducted using R software (v4.3.1).

## Results

### Identification of robust PCDSs in COAD

**Figure 2:**
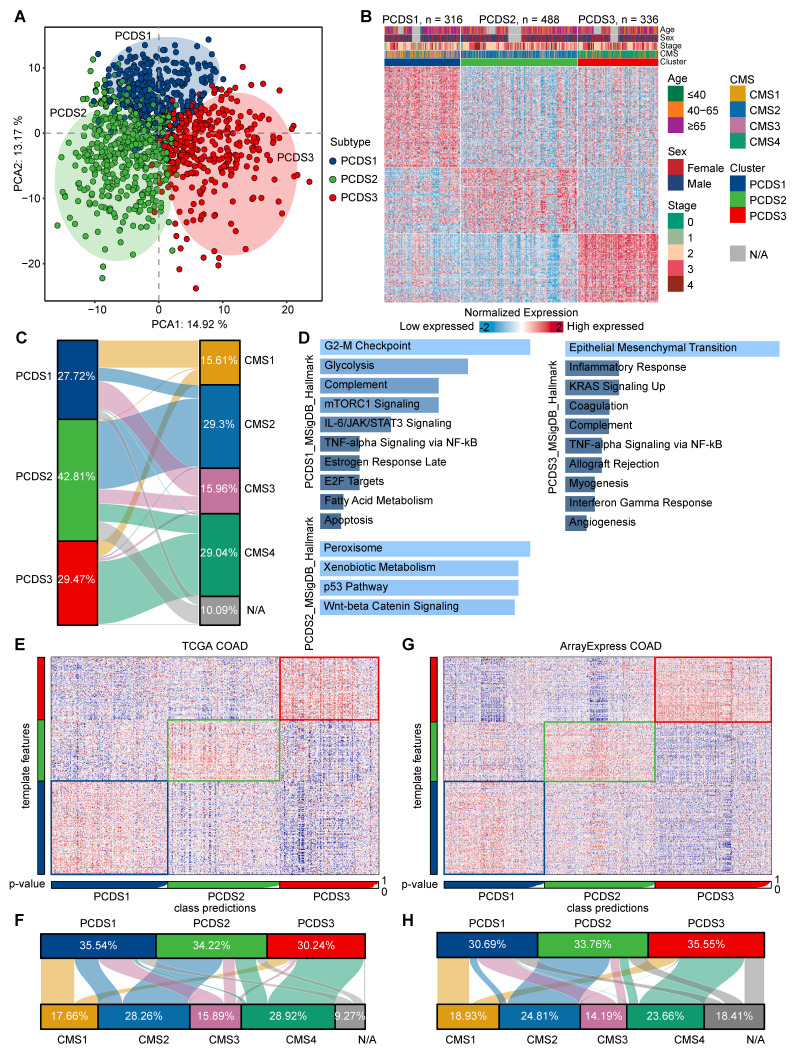
** The definition and robustness verification of PCDS. (A)** Principal component analysis (PCA) of 1,140 COAD patients based on 303 signature genes delineates three spatially distinct PCDSs. Each point represents a sample, and the color of the points corresponds to different PCDS.** (B)** Transcriptomic and clinicopathological landscape of PCDS in 1,140 COAD patients.​​ Heatmap displays normalized gene expression profiles across three PCDS groups (PCDS1, n = 316; PCDS2, n = 488; PCDS3, n = 336), with rows representing genes and columns representing individual samples. Key clinicopathological annotations include age, sex, tumor stage and Consensus Molecular Subtypes.** (C)** Sankey diagram showing flow distribution between PCDS and CMS in 1,140 COAD patients.** (D)** Functional enrichment analysis of differentially up-regulated genes in three PCDSs patients. MSigDB, Molecular Signatures Database.** (E, G)** Heatmap of the template feature expression level between 3 PCDSs in the TCGA **(E)** and ArrayExpress **(G)** COAD cohorts.** (F, H)** Sankey diagram showing flow distribution between PCDS and CMS in the TCGA **(F)** and ArrayExpress **(H)** COAD cohorts.

To capture coordinated dysregulation patterns of PCD pathways across COAD, we employed NMF to stratified 1,140 patients (referred to as GEO-COADs) based on PCD related gene list (**Table S1**) 42, which revealed three reproducible molecular subtypes. The selected number of clusters (k = 3) was determined by maximizing the cophenetic correlation coefficient and silhouette width across multiple runs (k = 3-7), ensuring robust and stable subtype identification (**Figure S1A-B**). Through subsequent filtering for high weight and subtype specificity (see Methods), we obtained a refined set of 303 signature genes uniquely associated with each PCDS (**Table S2**). These subtype-specific gene signatures delineated three clearly separated patient groups at the transcriptomic level, supporting the biological distinctiveness of each PCDS (**Figure 2A**). The expression patterns of 303 signature genes across three PCDSs and the corresponding clinical features of patients were displayed in the heatmap (**Figure 2B**). Comparison with the established CMS framework revealed both overlap and divergence. It showed that PCDS1 was composed of patients from diverse CMS subtypes, over half (56.15%) of the patients in PCDS2 were classified as CMS2, and the majority of patients in PCDS3 (75%) overlapped with the CMS4 patients (**Figure 2C**). Functional enrichment analysis of the differentially upregulated genes specific to each PCDS (**Table S3-5**) revealed distinct cancer hallmark associations: upregulated genes in PCDS1 were primarily associated with hallmarks such as 'G2-M Checkpoint', 'Glycolysis', 'mTORC1 Signaling', and 'IL-6/JAK/STAT3 Signaling'; those in PCDS2 were enriched for 'Peroxisome', 'Xenobiotic Metabolism', 'p53 Pathway', and 'Wnt-beta Catenin Signaling'; while upregulated genes in PCDS3 were predominantly enriched for 'Epithelial Mesenchymal Transition' and 'Inflammatory Response' (**Figure 2D**). Next, using the 303 signature genes defining three PCDSs, we performed the NTP (as detailed in Methods) on RNA-seq expression data from two distinct COAD cohorts sourced from ArrayExpress and TCGA respectively. The results of subtype prediction were shown in **Figure 2E** and **Figure 2G**, respectively. We also compared the patient proportions of PCDS and CMS classifications within these two validation datasets (**Figure 2F, H**), which revealed patterns similar to those observed in the microarray-based classification (**Figure 2C**), indicating that the PCDS classification was a robust subtype framework.

### Characterize PCDS associated genetic alterations and clinical prognosis

**Figure 3:**
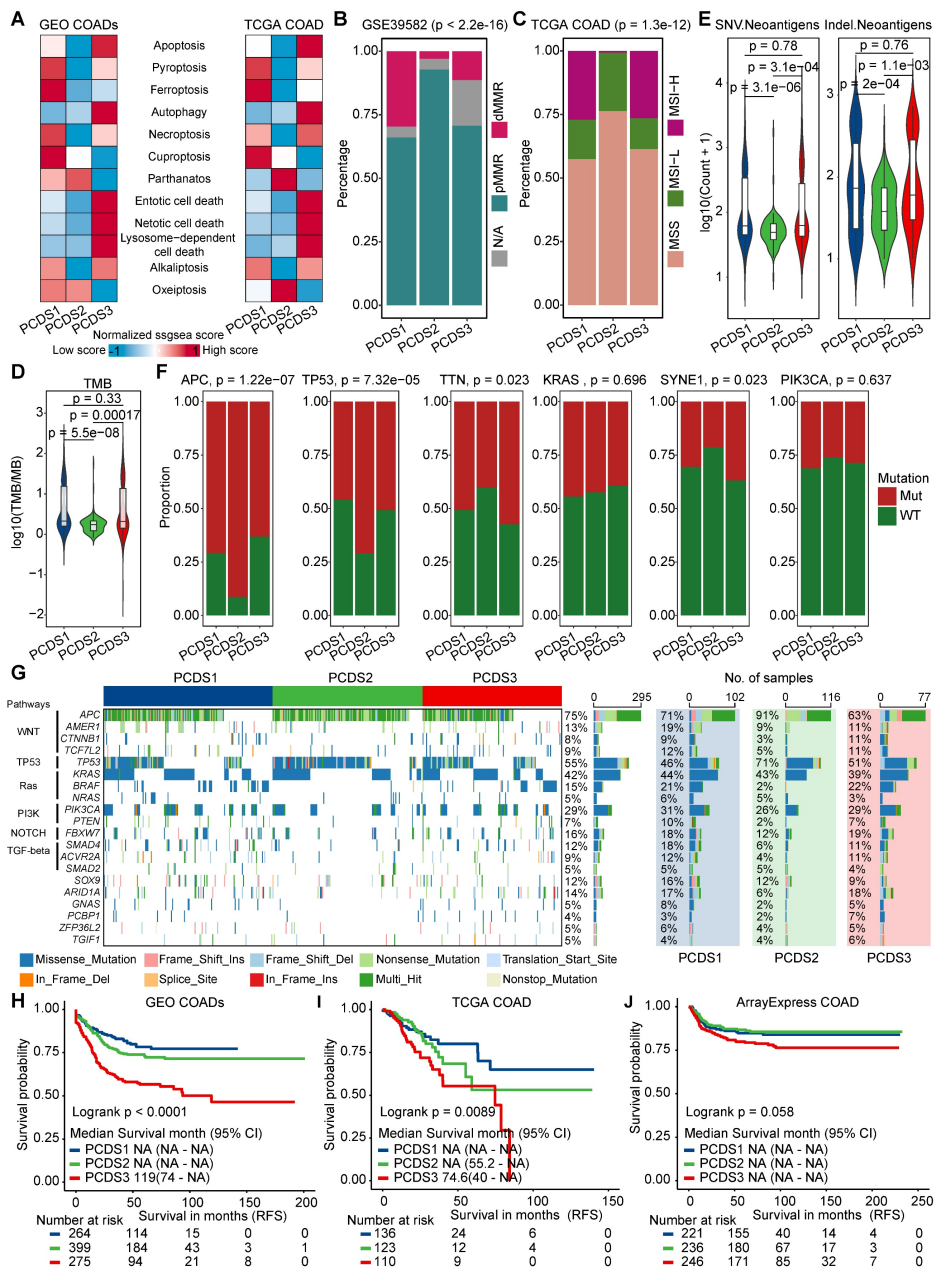
** Characterization of the genetic characteristics and clinical outcomes of PCDS. (A)** The heatmap showing differential activation of 12 PCD pathways across 3 PCDSs in GEO and TCGA COAD cohorts.** (B)** Stacked bar plot demonstrating the proportion of mismatch repair (MMR) status within three PCDSs from the GSE39582 cohort. P value was obtained by the χ² test (p values also apply to **C** and **F**).** (C)** Stacked bar plot demonstrating proportion of microsatellite instability (MSI) status within three PCDSs from the TCGA COAD cohort.** (D)** Comparison of tumor mutational burden (TMB) between three PCDSs from the TCGA COAD cohort. P value was obtained by the Wilcoxon rank-sum test (p values also apply to **E**).** (E)** Comparison of SNV- and Indel-derived neoantigens between three PCDSs from the TCGA COAD cohort.** (F)** Stacked bar plots demonstrating mutation frequency for six key driver genes (APC, TP53, TTN, KRAS, SYNE1, PIK3CA) within three PCDSs from the TCGA COAD cohort.** (G)** Oncoplot of pathway-centric gene mutational profiles across three PCDSs from the TCGA COAD cohort (left). The total mutation frequency of each gene and its mutation frequency in different PCDS patients (right).** (H-J)** Relapse-free survival (RFS) stratification by PCDS across three independent COAD cohorts. P values were obtained by the log rank test.

Each PCDS exhibited different patterns of PCD pathway activation (**Figure S2A-B** and **Figure 3A**), reflecting different cell-death regulatory states among three subtypes. Since genomic alterations frequently underlie transcriptional difference, we subsequently examined genomic aberrations across three PCDSs in multiple dimensions. First, we identified significantly different distributions of both mismatch repair (MMR) gene expression status (χ² test, *P* < 2.2e-16) and MSI status (χ² test, *P* = 1.3e-12) among three PCDSs (**Figure 3B-C**). Specifically, PCDS2 patients exhibited a higher level of proficient MMR (pMMR) expression, with MSI status primarily characterized by microsatellite stability (MSS) or low-frequency instability (MSI-L).

We examined the relationship between PCDS and driver gene mutations using TCGA COAD mutation data, initially characterizing the top 50 frequently mutated genes (**Figure S3A**). The TMB calculated from overall mutation profiles revealed significantly higher TMB in PCDS1 and PCDS3 compared to PCDS2 patients (**Figure 3D**). Meanwhile, PCDS1 and PCDS3 tumors showed significantly higher neoantigen loads (**Figure 3E**), suggesting greater intrinsic immunogenic potential than PCDS2. Focusing on six major driver genes, we observed significant differences in mutation frequencies for *APC* (χ² test, *P* = 1.22e-7), *TP53* (χ² test, *P* = 7.32e-5), *TTN* (χ² test, *P* = 0.023), and *SYNE1* (χ² test, *P* = 0.023) among three PCDSs (**Figure 3F**), with notably higher *APC* mutation frequency in PCDS2 versus lowest in PCDS3, while *TP53* mutations enriched in PCDS2 with similar frequencies in PCDS1 and PCDS3. Further characterization of six pathway-associated genes revealed infrequent mutations in PCDS2 patients (*CTNNB1*: 3%; *TCF7L2*: 5%; *BRAF*: 2%; *PTEN*: 2%; *SMAD4*: 6%; *ARID1A*: 6%; **Figure 3G**), with low *BRAF* mutation frequency aligning with MSS/MSI-L status 63. Given that genetic alterations impact survival outcomes, we conducted survival analyses using RFS as the primary clinical endpoint across all cohorts. Analysis of the integrated microarray cohort (GEO-COADs) and two independent RNA-seq cohorts (TCGA and ArrayExpress) confirmed significant RFS differences among three PCDSs, with PCDS3 consistently demonstrating the poorest prognosis (**Figure 3H-J**). This prognostic disadvantage of PCDS3 was further confirmed across three independent COAD datasets (GSE17538, GSE33113, and GSE39582) (**Figure S3B-D**).

### Determine PCDS specific variation in chemo- and immunotherapy responses

**Figure 4:**
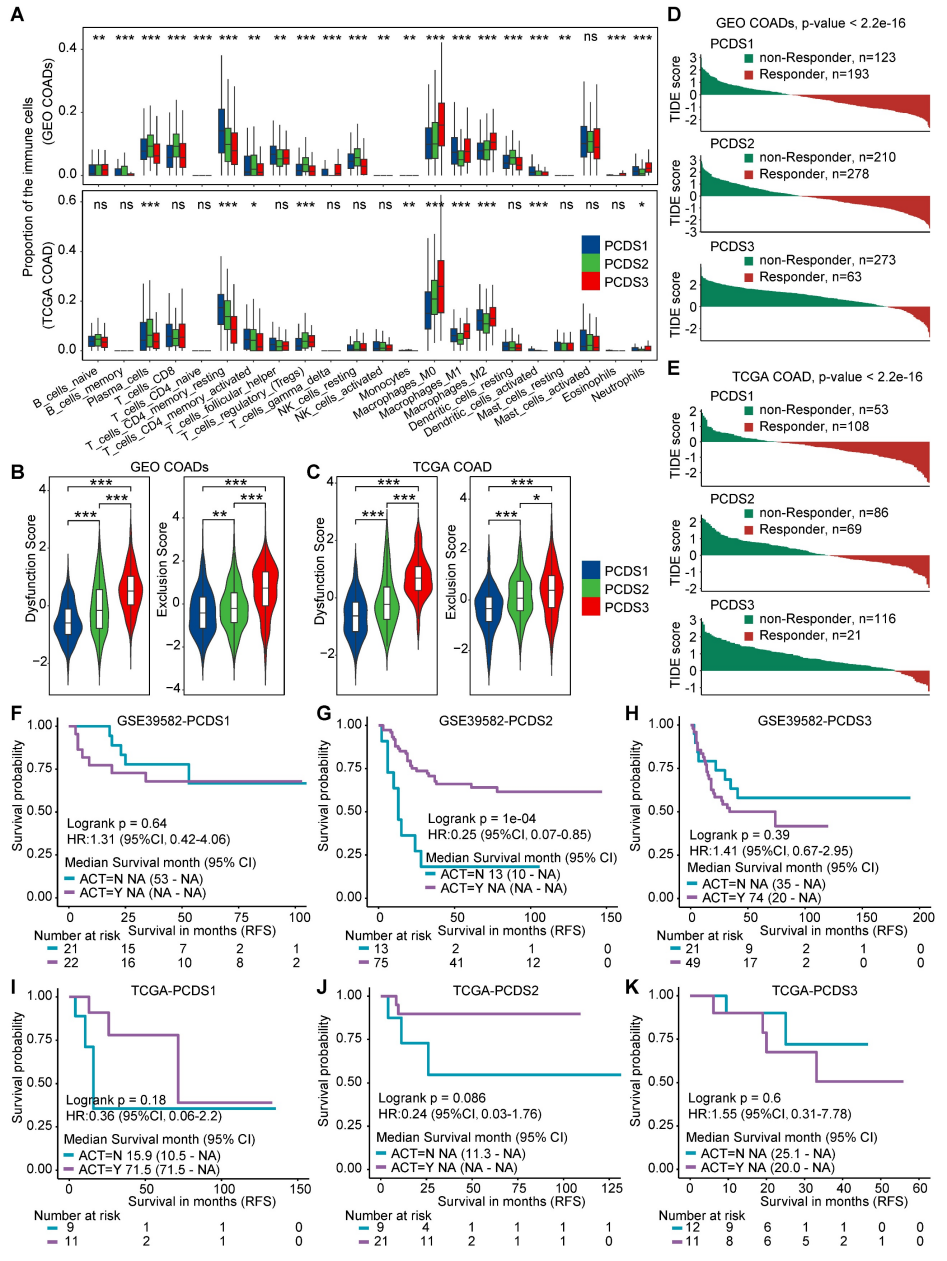
** The differential responses of PCDS to chemo/immuno-therapy. (A)** Box plots demonstrating differential immune cell infiltration landscape across three PCDSs in GEO and TCGA COAD cohorts. P values were calculated via one-way analysis of variance.** (B-C)** Comparison of T-cell dysfunction and exclusion scores in GEO **(B)** and TCGA **(C)** COAD Cohorts Stratified by PCDS​. P value was obtained by the Wilcoxon rank-sum test; ***, p <= 0.001; **, p <= 0.01; *, p <= 0.05; ·, p <= 0.1; ns, p > 0.1.** (D-E)** Waterfall plots showing immunotherapy response association with PCDS in GEO **(D)** and TCGA **(E)** COAD Cohorts. P value was obtained by the χ² test.** (F-H)** Relapse-free survival (RFS) demonstrating differential benefit from adjuvant chemotherapy (ACT) across PCDSs in the GSE39582 COAD cohort.** (I-K)** Relapse-free survival (RFS) demonstrating differential benefit from adjuvant chemotherapy (ACT) across PCDSs in the TCGA COAD cohort. P values were obtained by the log rank test. HR compares the RFS of the ACT-yes and ACT-no groups.

Immune deconvolution analysis revealed significant differences in immune cell composition across three PCDSs, particularly in T-cell and macrophage populations, implying the potential impact in immunotherapy response (**Figure 4A**). TIDE score is an effective tool of predicting ICB response 64, we observed markedly features of T-cell dysfunction and exclusion in PCDS3 patients (**Figure 4B-C**). To further explore the molecular basis of the different immunotherapy response, we analyzed the expression of key immune checkpoint and exhaustion markers across three PCDSs. In both the GEO and TCGA COAD cohorts, PCDS3 tumors consistently demonstrated significantly higher levels of immunosuppressive molecules, such as *PD-L1*, *PD-L2*, *CTLA-4*,* TIM-3*, *LAG-3*, and *TIGIT* (**Figure S4A-B**). This indicated that the PCDS3 is characterized by an extensive immunosuppressive environment. When patients were stratified by predicted immunotherapy benefit (**Figure 4D-E**), PCDS1 showed more responsive to immunotherapy (61.76% in GEO-COADs and 67.08% in TCGA-COAD), whereas PCDS3 represented an immune-refractory population (81.25% in GEO-COADs and 84.67% in TCGA-COAD), and PCDS2 patients showed no significant bias in immunotherapy response. Further, we applied the PCDS classifier to two independent cohorts (GSE235919 and GSE236581) (**Figure S4C-D**) where patients received ICB treatment. PCDS1 patients exhibited a higher complete and partial response rate and lower stable disease rate compared to PCDS3 patients (**Figure S4E-F**), which supported the association between PCDS1 and favorable immunotherapy response. Using RFS outcomes to assess ACT benefit 65, 66, we analyzed ACT-treated versus untreated subgroups in GSE39582 30 and TCGA COAD cohorts. Survival analyses indicated that ACT did not significantly improve RFS in either PCDS1 (**Figure 4F, I**) or PCDS3 patients (**Figure 4H, K**). Conversely, PCDS2 patients who received ACT showed longer RFS compared to those who did not (**Figure 4G, J**), suggesting a potential benefit of ACT in this subtype. Collectively, PCDS1 patients benefit from immunotherapy but not from ACT; PCDS2 patients benefit from ACT despite showing limited responsiveness to immunotherapy; while PCDS3 patients represent a treatment-resistant subpopulation that is unresponsive to both chemotherapy and immunotherapy.

### Single-cell analysis delineates TME differences across three PCDSs

**Figure 5:**
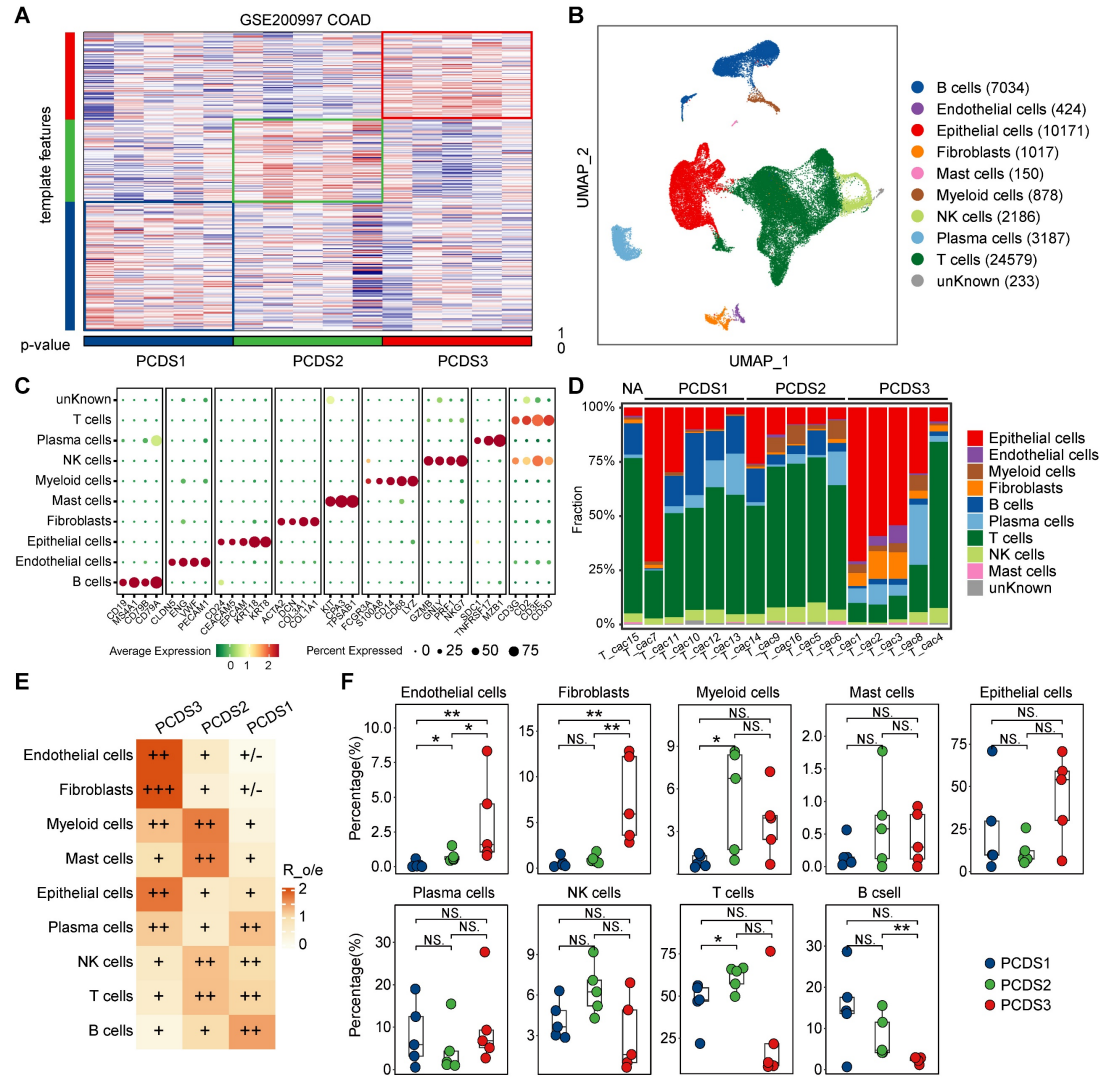
** Descriptions of the tumor microenvironment within different PCDS patients. (A)** Heatmap of the template feature expression level between 3 PCDSs in the GSE200997 COAD cohort.** (B)** Uniform Manifold Approximation and Projection (UMAP) plot showing 9 major cell types of n = 16 patients with COAD identified by integrated analysis. Each dot corresponds to a single cell, colored by cell types. NK cells, natural killer cells.** (C)** Dot plot showing average expression of known markers in indicated cell types. The dot size represents percent of cells expressing the genes in each cluster. The expression intensity of markers is shown.** (D)** Proportion of each major cell type in different PCDS patient, colored by cell types.** (E)** Relative subtype preference of each major cell type in different PCDS patient estimated by Ro/e.** (F)** Comparison of the infiltration abundances of 9 major cell types in three PCDSs. P value was obtained by the Wilcoxon rank-sum test; ***, p <= 0.001; **, p <= 0.01; *, p <= 0.05; ·, p <= 0.1; ns, p > 0.1.

Using paired bulk RNA-seq expression profiles from COAD patients in GSE200997 36, we applied the NTP algorithm to classify them into three PCDSs, with five patients in each subtype (**Figure 5A**). The processed scRNA-seq dataset consisting of 7 adjacent normal and 16 tumor tissues yielded 49,859 high-quality single cells. Single-cell analysis provided high-resolution insights into the TME, revealing different cellular composition across patients. We identified 38 distinct cell clusters, which were visualized using uniform manifold approximation and projection (UMAP) to depict their distribution across samples and tissue types (**Figure S5A**). Subsequent lineage-specific marker annotation defined each cluster's identity (**Figure S5B**).

The cells were categorized into 9 major cell types (**Figure 5B-C**), including B cells (n = 7034) marked by *CD19*, *MS4A1*, *CD79A* and *CD79B*, endothelial cells (n = 424) identified by the expression of *PECAM1*, *VWF*, *ENG* and *CLDN5*, epithelial cells (n = 10171) marked by *CD24*, *CEACAM5*, *EPCAM*, *KRT18* and *KRT8*, fibroblasts (n = 1017) defined by the classical markers of *ACTA2*, *DCN*, *COL3A1* and *COL1A1*, mast cells (n = 150) marked by *KIT*, *CPA3* and *TPSAB1*, myeloid cells (n = 878) identified by the expression of *FCGR3A*, *S100A8*, *CD14*, *CD68* and *LYZ*, NK cells (n = 2186) marked by *GZMB*, *GNLY*, *PRF1* and *NKG7*, plasma cells (n = 3187) marked by *SDC1*, *TNFRSF17* and *MZB1*, and T cells (n = 24579) which expressed *CD3G*, *CD2*, *CD3E* and *CD3D*. Although all 9 cell types were present in tumor tissues across three PCDSs (**Figure 5D**), differential infiltration hierarchies of these cell types signified inter-subtype variance in the cellular architecture of the TME. We subsequently quantified relative PCDS enrichment of main cell clusters by calculating the ratio of observed to expected cell numbers (Ro/e) using data of patients with PCDS label (**Figure 5D-E**), the result demonstrated obvious enrichment of endothelial cells and fibroblasts in PCDS3 patients. Furthermore, we calculated the absolute proportions of 9 cell types in each patient, which revealed consistent results supporting the observed hierarchical patterns (**Figure 5F**). We next analyzed an additional scRNA-seq dataset (GSE178341) comprising 62 colorectal cancer patients. Pseudobulk expression profiles were generated and subjected to PCDS classification, yielding 27 PCDS1, 19 PCDS2, and 16 PCDS3 patients (**Figure S6A-B**). Notably, the cellular composition across PCDSs showed a significant enrichment of endothelial cells and fibroblasts in PCDS3 tumors, thus independently confirming the fibroblast enriched microenvironment characteristic of PCDS3 (**Figure S6C**).

### Cell-cell interaction of tumor cells and fibroblasts revealed the potential therapeutic target for PCDS3 patients

**Figure 6:**
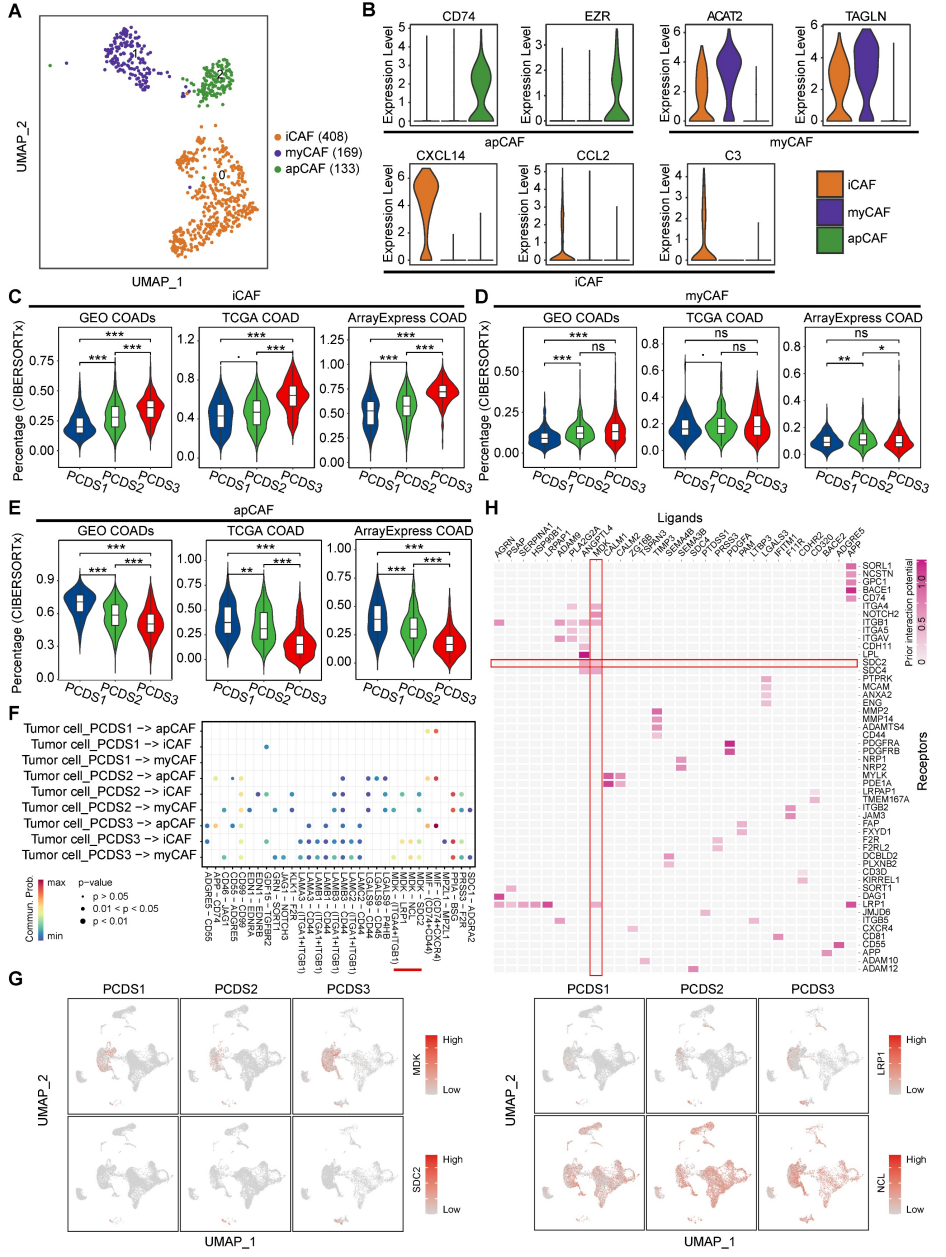
** Characterization of fibroblasts in different PCDSs. (A)** UMAP plot of individual fibroblasts. Each dot denotes one cell; color represents subcluster. iCAF, inflammatory CAF; myCAF, myofibroblasts; apCAF, antigen presenting CAF.** (B)** Violin plot of selected apCAF markers (CD74 and EZR), myCAF markers (ACAT2 and TAGLN), and iCAF markers (CXCL14, CCL2 amd C3) showing normalized expression in each of the subclusters.** (C-E)** Comparison of the infiltration abundances of 3 CAF subtypes across PCDSs in multi-cohort COAD analysis, including COAD cohorts from GEO, TCGA, and ArrayExpress. P value was obtained by the Wilcoxon rank-sum test; ***, p <= 0.001; **, p <= 0.01; *, p <= 0.05; ·, p <= 0.1; ns, p > 0.1.** (F)** Dot plot showing the ligand-receptor pairs in the communication of three types of PCDS tumor cells to three types of fibroblasts. P values are indicated by circle size and communication probabilities are indicated by circle color.** (G)** UMAP plots showing the expression of selected ligand (MDK) and three receptors (SDC2, LRP1 and NCL) among 9 major cell types. Each dot corresponds to a single cell. Darker color represents higher gene expression. (**H**) Ligand-receptor pairs showing interaction between tumor cells and CAFs in PCDS3 subtype, as predicted by NicheNet software.

Fibroblast enrichment in PCDS3 patients potentially enables more complex tumor-stroma crosstalk. We employed CopyKAT to identify 2,568 malignant tumor cells from 10,171 epithelial cells (**Figure S7A**), subsequently subclustering fibroblasts into three subclusters (**Figure S7B**) that were annotated via established lineage-defining markers 67 as myofibroblasts (myCAF), inflammatory CAF (iCAF), and antigen presenting CAF (apCAF) (**Figure 6A-B**). Based on the distinct characteristics of these three fibroblast types (**Table S6**), we used the CIBERSORTx (https://cibersortx.stanford.edu/) to estimate their relative infiltration abundance across three COAD cohorts. The results consistently showed that iCAF had the highest infiltration in PCDS3 patients, while apCAF was most abundant in PCDS1 patients, potentially explaining the better immunotherapy response in PCDS1 patients. The infiltration of MyCAF showed no significant differences among the three PCDSs (**Figure 6C-E**). CellChat was employed to identify the ligand-receptor pairs involved in interactions between tumor cells and fibroblasts across three PCDSs. We found that tumor cells exhibited stronger MDK-based interactions with iCAF or myCAF in PCDS3 patients (**Figure 6F**). **Figure 6G** indicated that the ligand *MDK* is specifically expressed in tumor cells. Among the three receptors (*SDC2*, *LRP1*, and *NCL*), only *SDC2* is specifically expressed in fibroblasts, suggesting that tumor cells in PCDS3 patients interact with iCAF/myCAF via the MDK-SDC2 ligand-receptor pair. Furthermore, using NicheNet to compare PCDS3 with PCDS1 and PCDS2, we observed that MDK-SDC2 exhibited significant ligand-receptor activity in PCDS3 (**Figure 6H**), confirming the active signaling role of the MDK-SDC2 axis in this subtype. To validate the MDK-SDC2 interaction, ST data from 4 COAD patients were analyzed (**Figure S7C-F**). The results demonstrated spatial co-localization of *MDK* and *SDC2* expression. Specifically, in ST-colon2 and ST-colon3 patients, regions with high T-cell infiltration exhibited lower expression levels of both *MDK* and *SDC2* (**Figure S7D-E**). These findings suggested that the interaction between tumor cells and iCAF/myCAF via the MDK-SDC2 axis in PCDS3 patients may be associated with T-cell exclusion. Subsequently, in two COAD cohorts, samples were divided into four groups according to the median expression levels of *MDK* and *SDC2*. Notably, TIDE analysis revealed higher scores for T-cell dysfunction and exclusion in the *MDK*-high/*SDC2*-high group (**Figure S7G-H**).

**Figure 7:**
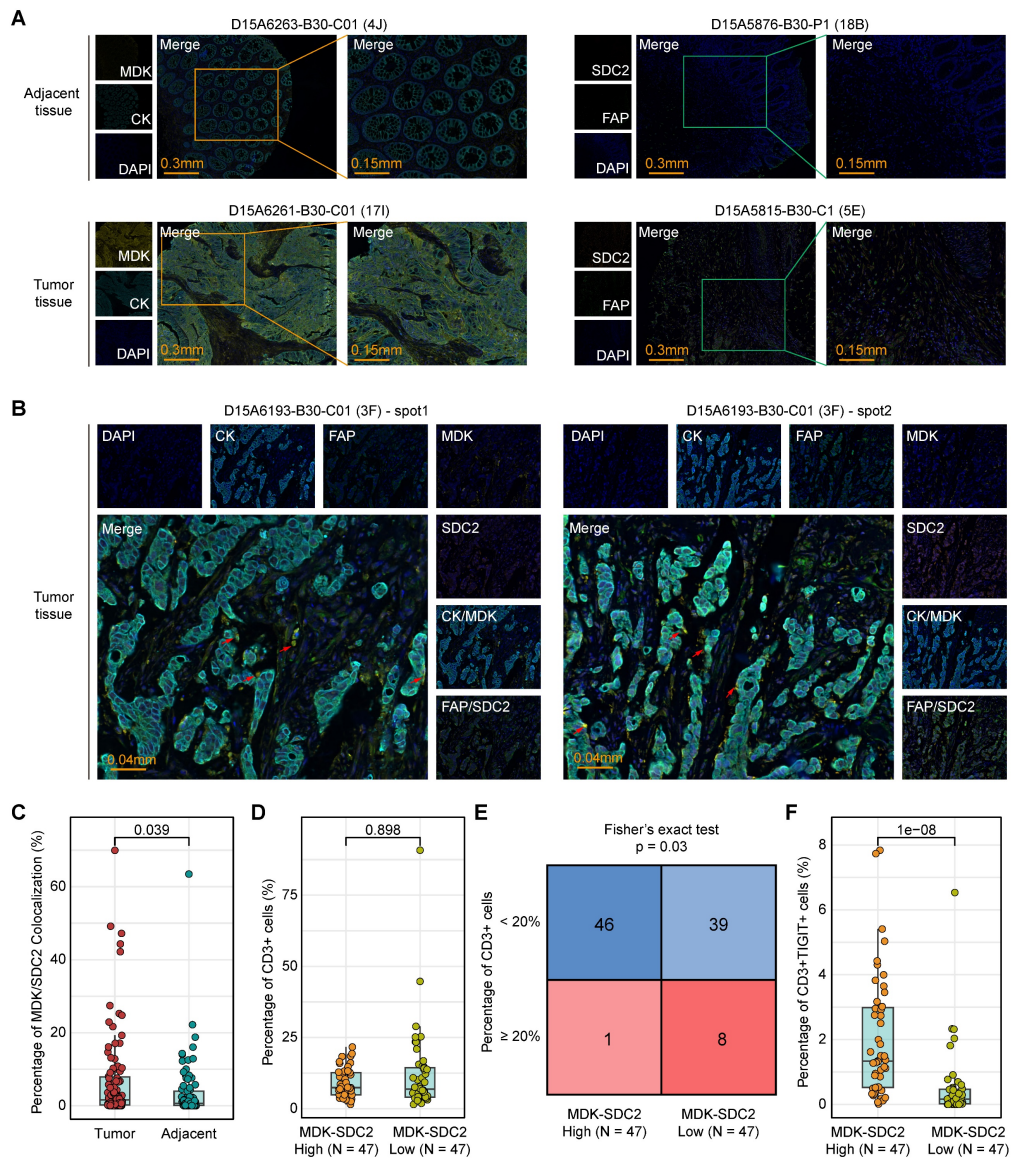
** Association between MDK-SDC2 mediated tumor-fibroblast interactions and T-cell dysfunction and exclusion. (A-B)** Representative mIF images showing CK, MDK, FAP and SDC2 in different tissues.** (C)** Comparison of the co-localization ration of tumor cell-derived MDK and fibroblast-derived SDC2 within tumor regions. P value was obtained by the Wilcoxon rank-sum test (p values also apply to D and F).** (D)** Comparison of the ration of CD3⁺ T cells between MDK-SDC2 co-localization-high and -low groups.** (E)** The sample distribution with a threshold of 20% infiltration ratio of CD3⁺ T cells between MDK-SDC2 co-localization-high and -low groups. **(F)** Comparison of the ration of TIGIT⁺ exhausted T cells between MDK-SDC2 co-localization-high and -low groups.

We next sought orthogonal experimental validation at the tissue level. The mIF staining of tumor tissues from 94 colorectal cancer patients and matched adjacent tissues from 86 cases (**Table S7**) confirmed the spatial co-localization of tumor cell-derived MDK and fibroblast-derived SDC2 within tumor regions (**Figure 7A-B**). Importantly, MDK-SDC2 co-localization was significantly more frequent in tumor tissues than in adjacent tissues (Wilcoxon rank-sum test, *P* = 0.039; **Figure 7C**). To assess the immunological relevance of this tumor-fibroblast interaction, 94 tumor samples were stratified into MDK-SDC2 co-localization-high and -low groups based on the median co-localization ratio. While the overall abundance of CD3⁺ T cells was similar in the two groups (Wilcoxon rank-sum test, *P* = 0.898; **Figure 7D**), tumors with low MDK-SDC2 co-localization were significantly enriched for cases with high CD3⁺ T-cell infiltration when a 20% cutoff was applied (Fisher's exact test, *P* = 0.03; **Figure 7E**). And tumors with low MDK-SDC2 co-localization also exhibited a markedly reduced proportion of TIGIT⁺ exhausted T cells (Wilcoxon rank-sum test, *P* = 1e-08; **Figure 7F**). Collectively, these findings provide convergent evidence supporting an association between MDK-SDC2-mediated tumor-fibroblast interactions and T-cell dysfunction and exclusion.

### Targeting SDC2 with Sunitinib represents a potential therapeutic strategy for PCDS3 patients

**Figure 8:**
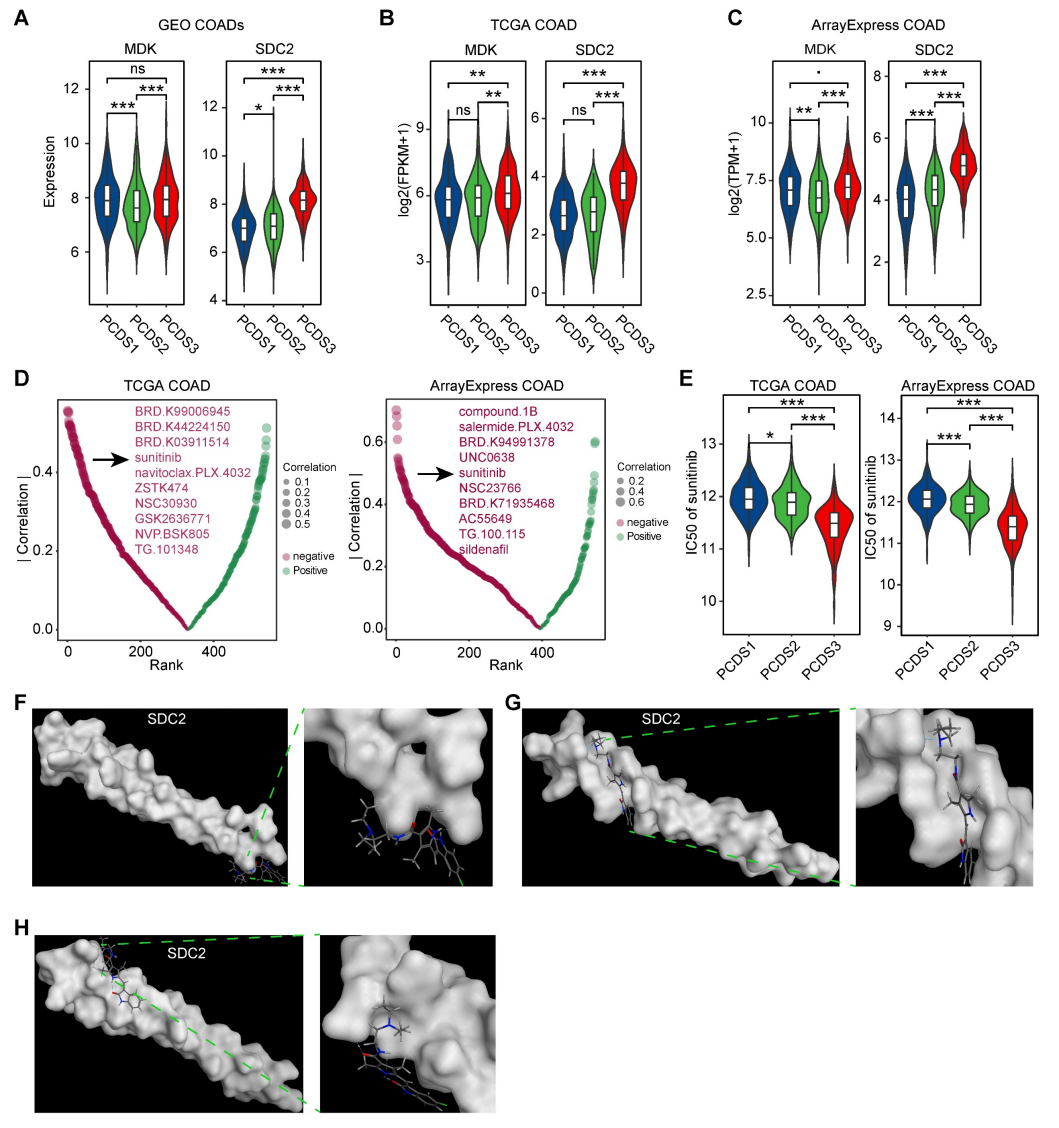
** Identification of potential targeted drugs in PCDS3 patients. (A-C)** Comparison of the expression of selected ligand (MDK) and receptor (SDC2) across 3 PCDSs in GEO **(A)**, TCGA **(B)**, and ArrayExpress **(C)** COAD cohorts. P value was obtained by the Wilcoxon rank-sum test (p values also apply to E); ***, p <= 0.001; **, p <= 0.01; *, p <= 0.05; ·, p <= 0.1; ns, p > 0.1.** (D)** Correlation between SDC2 expression and drug sensitivity profiles across COAD cohorts from TCGA and ArrayExpress. Each dot denotes one drug; size of dot represents the degree of correlation; color represents the direction of correlation.** (E)** Comparison of the differential sensitivity to sunitinib across three PCDSs in TCGA and ArrayExpress COAD cohorts.** (F-H)** Structural basis of SDC2-sunitinib binding interface revealed by molecular docking. The top three binding conformations exhibiting S-scores of -17.2199 **(F)**, -15.4535** (G)**, and -13.8831 **(H)**, respectively. Left panels showing panoramic views of each conformation complex; right panels showing magnified views of the binding interfaces.

Disrupting the MDK-SDC2 interaction between tumor cells and fibroblasts may offer new treatment opportunities for PCDS3 patients. We revalidated the expression levels of the ligand *MDK* and the receptor *SDC2* across three COAD cohorts and found that *SDC2* expression was consistently and significantly higher in PCDS3 patients compared to the other two subtypes (**Figure 8A-C**). We then used the “oncoPredict” R package to assess the association between *SDC2* expression and the IC50 of several anticancer drugs. As shown in **Figure 8D**, two independent analyses using COAD RNA-seq expression data identified the top 10 anticancer drugs whose IC50 values were significantly negatively correlated with *SDC2* expression. Among them, sunitinib was the only drug consistently identified across both datasets. Further analysis showed that the IC50 of sunitinib was significantly lower in PCDS3 patients (Wilcoxon rank-sum test, *P* < 0.001) compared to the other two subtypes (**Figure 8E**), indicating that sunitinib may have a relatively better anticancer effect in PCDS3 patients. To further validate the potential binding affinity between sunitinib and *SDC2*, we performed molecular docking using the MOE software. We identified multiple binding conformations between sunitinib and *SDC2*, as shown in **Figure 8F-H**, with the top three binding conformations exhibiting S-scores of -17.2199 (**Figure 8F**), -15.4535 (**Figure 8G**), and -13.8831 (**Figure 8H**), respectively, suggesting that sunitinib is a promising potential targeted drug for *SDC2*.

## Discussion

In this study, we developed a novel molecular classification system for COAD, named PCDS, by integrating 12 distinct PCD pathways. Using NMF, we identified three distinct biological subtypes across 1,140 patients, including PCDS1 (immune-activated), PCDS2 (WNT and TP53 signaling activation), and PCDS3 (mesenchymal and T-cell dysfunction/exclusion). PCDS1 is an immune activation subtype marked by a pro-inflammatory TME with metabolic activation and a high abundance of apCAFs, suggesting sustained immune recognition. These features likely explain its responsiveness to ICB. Activation of the G2-M checkpoint and mTORC1 pathways reflects intrinsic resistance mechanisms that maybe reduce the efficacy of chemotherapy. PCDS2 shows an epithelial phenotype driven by canonical APC and TP53 mutations and metabolic pathways such as 'Peroxisome' and 'Xenobiotic Metabolism'. These characteristics likely explain the subtype's vulnerability to cytotoxic drugs, suggesting that DNA-damaging chemotherapy and pathway-targeted therapies could be particularly effective. PCDS3 is a mesenchymal, stromal-dominant subtype marked by extensive fibroblast activation and endothelial expansion. The significant enrichment of EMT and inflammatory pathways reflects a more complex TME, where tumor-derived MDK interacts with fibroblast-derived SDC2, potentially promoting T-cell dysfunction/exclusion. Together with increased immune exhaustion and suppressive signatures, these features define the immunosuppressive phenotype of PCDS3. This phenotype may explain why PCDS3 tumors remain resistant to immunotherapy despite having high levels of MSI, TMB burden, and neoantigen load. Accordingly, PCDS3 represents a stroma-enriched and immune-excluded state that may require therapeutic strategies targeting the tumor stroma. In our study, identifying sunitinib as a potential SDC2-targeting agent provides a potential rational approach to alleviate stromal barriers and enhance immune response in PCDS3 tumors.

We evaluated the therapeutic responses across three subtypes, in which PCDS1 patients responded to immunotherapy but showed limited benefit from ACT; PCDS2 patients benefited from ACT; and PCDS3, overlapping with CMS4 in 75% of cases, was a dual-resistant subtype, with over 80% cases showing no response to immunotherapy and no survival benefit from ACT. While the CMS 11 framework has provided an invaluable foundation for understanding the biological heterogeneity of colorectal cancer, our PCDS classification offers a complementary perspective that could help decipher clinically therapy resistance. PCD pathways are key regulators of therapeutic response, influencing sensitivity to chemotherapy, targeted therapy, and immunotherapy 68-72. By systematically linking dysregulated PCD pathways to TME remodeling and therapeutic resistance, the PCDS framework offers a clear understanding of clinical outcomes and resistance patterns. Previous studies have shown that high levels of MSI-H, TMB, and neoantigens correlate with better immunotherapy responses 73, 74. Notably, the PCDS framework identifies different immunophenotypes in tumors with high MSI, TMB, and neoantigen burden, in which PCDS1 shows strong immunotherapy response, while PCDS3 remains unresponsive despite having similar MSI, TMB, and neoantigen levels. PCDS3 further reveals a stromal-dominated microenvironment characterized by extensive fibroblast activation and endothelial expansion, linked to the MDK-SDC2 signaling axis between tumor cells and CAFs. This interaction may create a stromal barrier in PCDS3 tumors, promoting T-cell dysfunction and exclusion in the MSI-high tumors, and explaining the lack of response to ICB treatment. MDK signaling has been implicated in creating an immunosuppressive niche by recruiting regulatory cell types and modulating chemokine, which could spatially limit T-cell infiltration 75, 76. Despite strong associations supported by data driven analyses and mIF staining, a key limitation of this study is the lack of direct functional evidence linking the MDK-SDC2 axis to T-cell dysfunction/exclusion. Disrupting this axis may restore T-cell activity and resensitize PCDS3 tumors to immunotherapy, a hypothesis to be tested in future co-culture models and genetic perturbation experiments. To explore this hypothesis, we employed drug sensitivity prediction tools and the GDSC2 database, identifying sunitinib as a potential SDC2-targeting agent. Molecular docking confirmed its strong binding affinity to SDC2. Sunitinib, a multi-targeted receptor tyrosine kinase (RTK) inhibitor, blocks key signaling pathways involved in tumor growth and angiogenesis 77, 78. Clinically, it is a first-line treatment for advanced or metastatic clear cell renal cell carcinoma, significantly improving progression-free survival (PFS: 11 vs. 5 months) and overall survival (OS: 26.4 vs. 21.8 months) compared to interferon-α, with an objective response rate (ORR) of 53% 78, 79. As a second-line therapy for imatinib-resistant or intolerant gastrointestinal stromal tumors (GIST), sunitinib extended the time to progression from 6.4 to 27.3 weeks (HR = 0.33), with an ORR of 7% compared to 0% in placebo 78, 80. However, sunitinib has not demonstrated meaningful clinical efficacy as monotherapy in unselected colorectal cancer populations, highlighting the need for subtype-specific and combination-based validation strategies.

Single-cell sequencing technologies, by resolving transcriptomic, epigenetic, or proteomic profiles at single-cell resolution, have overcome the limitations of bulk sequencing and enabled the identification of rare cellular subpopulations (e.g., specific fibroblast subsets) that constitute less than 1% of the total tissue 81-85. With the advancement of identification methods of tumor cells (e.g., inferCNV 86, CopyKAT 51, cancer-finder 87, SCANER 88), trajectory inference tools (e.g., Monocle 89, Slingshot 90, RNA Velocity 91) and cell-cell communication platforms (e.g., CellPhoneDB 92, CellChat 58, NicheNet 59), we can now trace lineage dynamics and decode intricate intercellular interactions within the TME. Our PCDS model was developed using large-scale bulk RNA-seq data. Leveraging the work of Ateeq M et al., who provided matched single-cell and bulk RNA-seq data for COAD patients 36, we were able to first classify patients using our bulk-based PCDS system and subsequently dissect the tumor ecosystem of each subtype at single-cell resolution. While we have also validated our key findings by applying the PCDS framework to pseudobulk gene expression profiles generated from two additional single-cell datasets (GSE178341 37 and GSE236581 38), future work will involve generating more matched bulk and single-cell RNA-seq data to further substantiate our conclusions.

Despite the strengths of our findings, this study has several limitations. In terms of sample representativeness, our retrospective analysis of GEO/TCGA datasets may be subject to selection bias, especially due to the underrepresentation of metastatic samples. Future prospective clinical trials are needed to validate the predictive value of the PCDS classification in advanced COAD. The assessment of differential ACT benefit across PCDSs is based on retrospective comparison of RFS in treated versus untreated subgroups, which is susceptible to treatment selection bias and confounding factors. While this approach is a recognized method for generating hypotheses in the absence of randomized trial data 65, 66, prospective validation is required to confirm the ACT benefit specifically in PCDS2 patients in the future. A key issue that also needs to be addressed in future studies is that our proposed link between the MDK-SDC2 axis and T-cell dysfunction/exclusion, although strongly supported by various results, still requires direct functional validation. To fill the mechanistic gaps in our study, we outline two focused directions for future validation. First, the functional role and upstream regulation of the MDK-SDC2 axis in PCDS3 patients will be investigated using patient-derived tumor organoids with CAFs and immune cell co-culture systems. Specifically, we will explore whether MDK overexpression or knockdown in tumor and SDC2 overexpression or knockdown in CAF affect T-cell infiltration, cytotoxicity, and exhaustion. These assays will determine if MDK-SDC2 signaling drives T-cell dysfunction/exclusion and whether disrupting this axis via SDC2 perturbation or pharmacological blockade can restore T-cell infiltration and activation. Complementary ChIP-seq profiling will identify transcriptional regulators of SDC2, potentially allowing us to reduce the downstream expression of SDC2 by inhibiting the key transcription factors (TFs), thus reducing the MDK-SDC2 interaction. Another key issue is to investigate the potential therapeutic role of sunitinib in targeting SDC2. We will combine CRISPR-mediated SDC2 knockout with humanized PDX model (hu-PDX) derived from PCDS3 patients. We will establish hu-PDX by implanting hematopoietic stem cells into immunodeficient mice, followed by tumor grafting from PCDS3 patients. We will compare tumor size, survival data, and the immune microenvironment in mice under three conditions, including unmodified hu-PDX, hu-PDX with CRISPR-Cas9-mediated SDC2 knockout, and hu-PDX treated with sunitinib, to determine whether SDC2 is a target of sunitinib. Additionally, PCDS3-derived hu-PDX models will also be treated with anti-PD-1 alone or combined with sunitinib to assess whether inhibiting SDC2 improves the response to ICB.

## Conclusion

Our study proposed a molecular subtype based on programmed cell death (PCD) that identifies distinct therapeutic phenotypes in COAD. Notably, we identified PCDS3 as a dual-resistant subtype characterized by a fibroblast-enriched and immune-excluded tumor microenvironment. Mechanistic analyses implicated MDK-SDC2 signaling in driving T cell dysfunction, and we proposed sunitinib as a potential stroma-targeted therapy. These findings underscored the clinical relevance of integrating cell death pathways with tumor microenvironment profiling and point to a tractable strategy for overcoming therapeutic resistance in COAD.

## Supplementary Material

Supplementary figures and tables.

## Figures and Tables

**Table 1:** Patients and tumor characteristics of the different datasets.

Characteristics	Type	Mean age (sd, range), years	Sex (male/female)	TNM stage						ACT(Yes/No)
				**0**	**I**	**II**	**III**	**IV**	**NA**	
GSE17538	mRNA, Microarray	64.7(13.4, 23-94)	122/110	0	27	72	76	56	0	/
GSE33113	mRNA, Microarray	70.4(13.1, 35-95)	42/48	0	0	90	0	0	0	/
GSE37892	mRNA, Microarray	68.3(12.7, 22-97)	69/61	0	0	73	57	0	0	/
GSE38832	mRNA, Microarray	/	/	0	18	35	39	30	0	/
GSE39582	mRNA, Microarray	66.9(13.3, 22-97)	310/256	4	33	264	205	60	0	233/316
TCGA	mRNA, RNA-seq	67.0(13.1, 31-90)	239/212	0	75	177	126	63	12	80/302
ArrayExpress	mRNA, RNA-seq	72.0(11.3, 30-94)	377/405	0	90	326	287	79	0	33/748
GSE200997	mRNA, scRNA-seq	/	/	/	/	/	/	/	/	/

ACT, Adjuvant chemotherapy. NA, not available; sd, standard deviation.

## Data Availability

All data used in this study are publicly available from previous publications. Additional information for data reanalysis can be obtained from the lead contact upon request.

## Abbreviations

COAD: colon adenocarcinoma
CMS: Consensus Molecular Subtypes
MSI: microsatellite instability
PCD: programmed cell death
TME: tumor microenvironment
CAF: cancer-associated fibroblast
EMT: epithelial-mesenchymal transition
PCDS: Programmed Cell Death-related Subtype
RFS: relapse-free survival
TCGA: The Cancer Genome Atlas
ICB: immune checkpoint blockade
ST: spatial transcriptome
fRMA: Frozen Robust Multi-array Analysis
NMF: non-negative matrix factorization
ssGSEA: single sample gene set enrichment analysis
NTP: Nearest Template Prediction
ACT: adjuvant chemotherapy
TMB: tumor mutational burden
TIDE: Tumor Immune Dysfunction and Exclusion
CCA: Canonical Correlation Analysis
DEGs: Differentially expressed genes
mIF: multiplex immunofluorescence
DAPI: 4,6-Diamidino-2-phenylindole
CK: pan-cytokeratin
IC50: the half maximal inhibitory concentration
GDSC2: Drug Sensitivity in Cancer 2
MOE: Molecular Operating Environment
PDB: Protein Data Bank
MMR: mismatch repair
MSS: microsatellite stability
UMAP: uniform manifold approximation and projection
RTK: receptor tyrosine kinase
PFS: progression-free survival
OS: overall survival
ORR: objective response rate
GIST: gastrointestinal stromal tumors
TFs: transcription factors
hu-PDX: humanized PDX model
