# Supplementary material for: Identification of programmed cell death-related subtypes reveals immune heterogeneity and therapeutic divergence in colon cancer
Source: Theranostics. 2026 Feb 18;16(9):4821–40. doi: 10.7150/thno.126314 (PMC12964230; doi:10.7150/thno.126314)
Supplement: Supplementary file 1 — Supplementary figures and tables. [file thnov16p4821s1.zip › Supplementary figures.pdf]

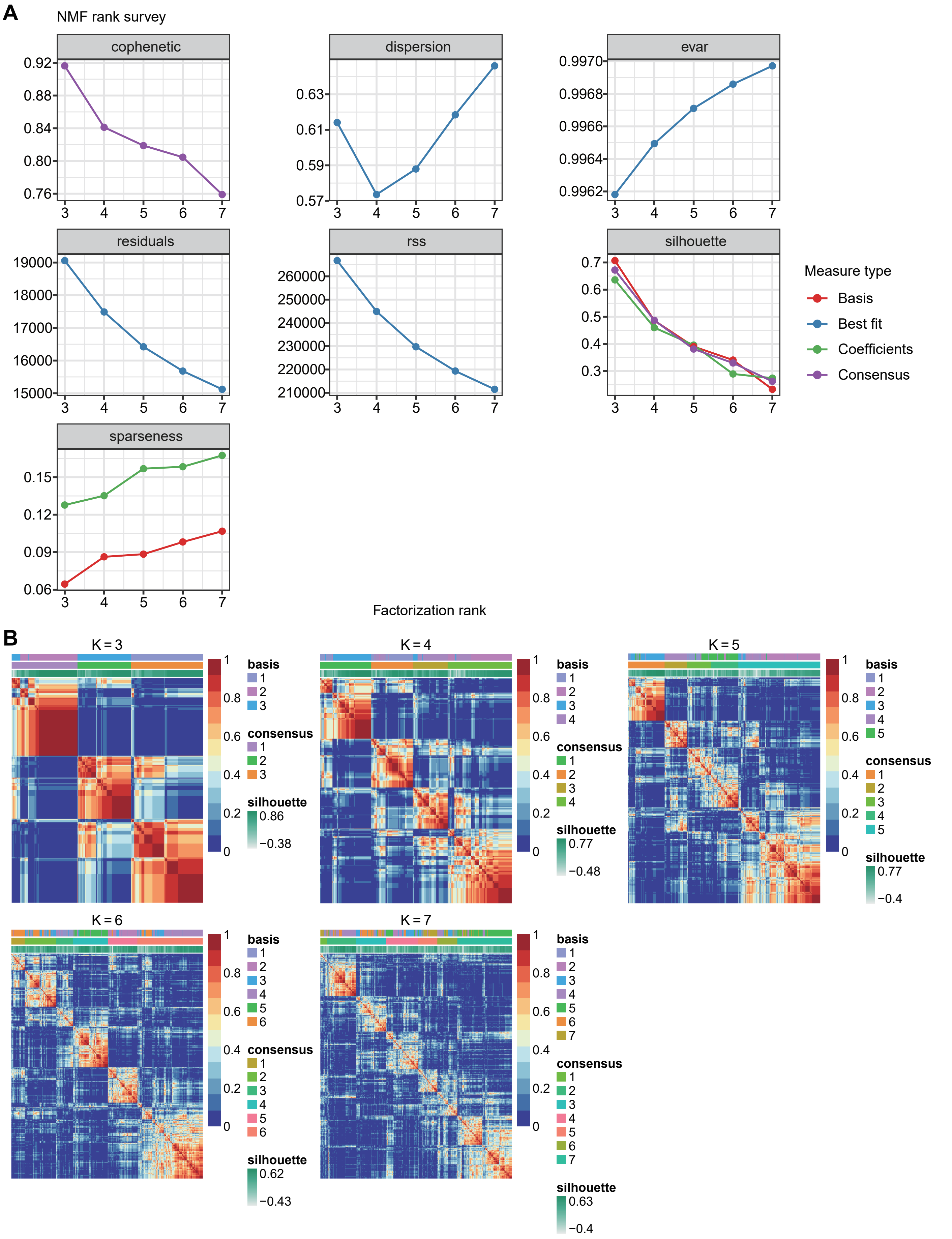

Figure S1. NMF clustering divided the samples of GEO-COADs cohort into distinct subtypes. (**A**) NMF clustering coefficients for k = 3-7. (**B**) Consensus map of NMF clustering for k = 3-7.

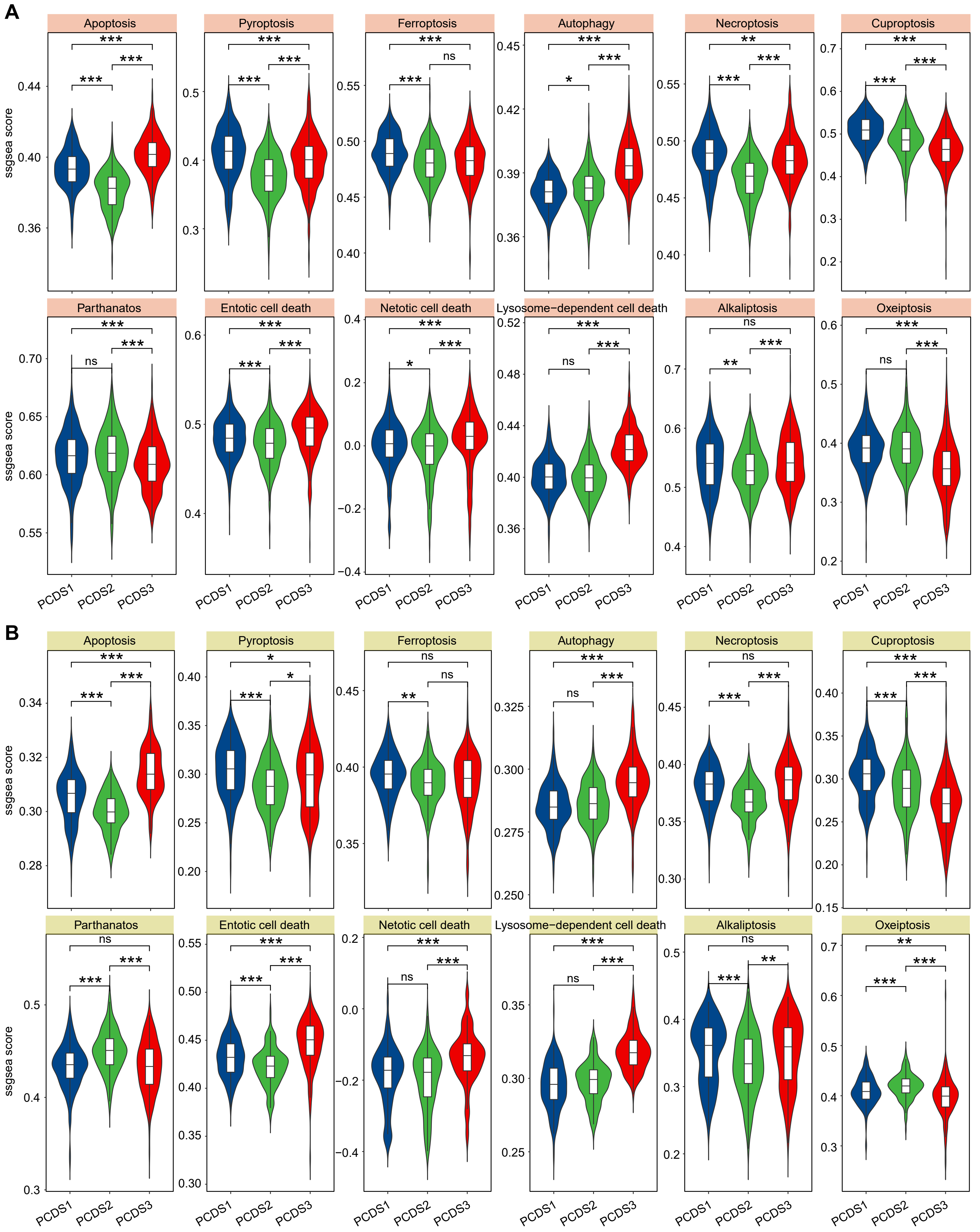

Figure S2. The activation scores of 12 PCD pathways in COAD patients form GEO (A) and TCGA (B). P value was obtained by the Wilcoxon rank-sum test; ***, p <= 0.001; **, p <= 0.01; *, p <= 0.05; ·, p <= 0.1; ns, p > 0.1.

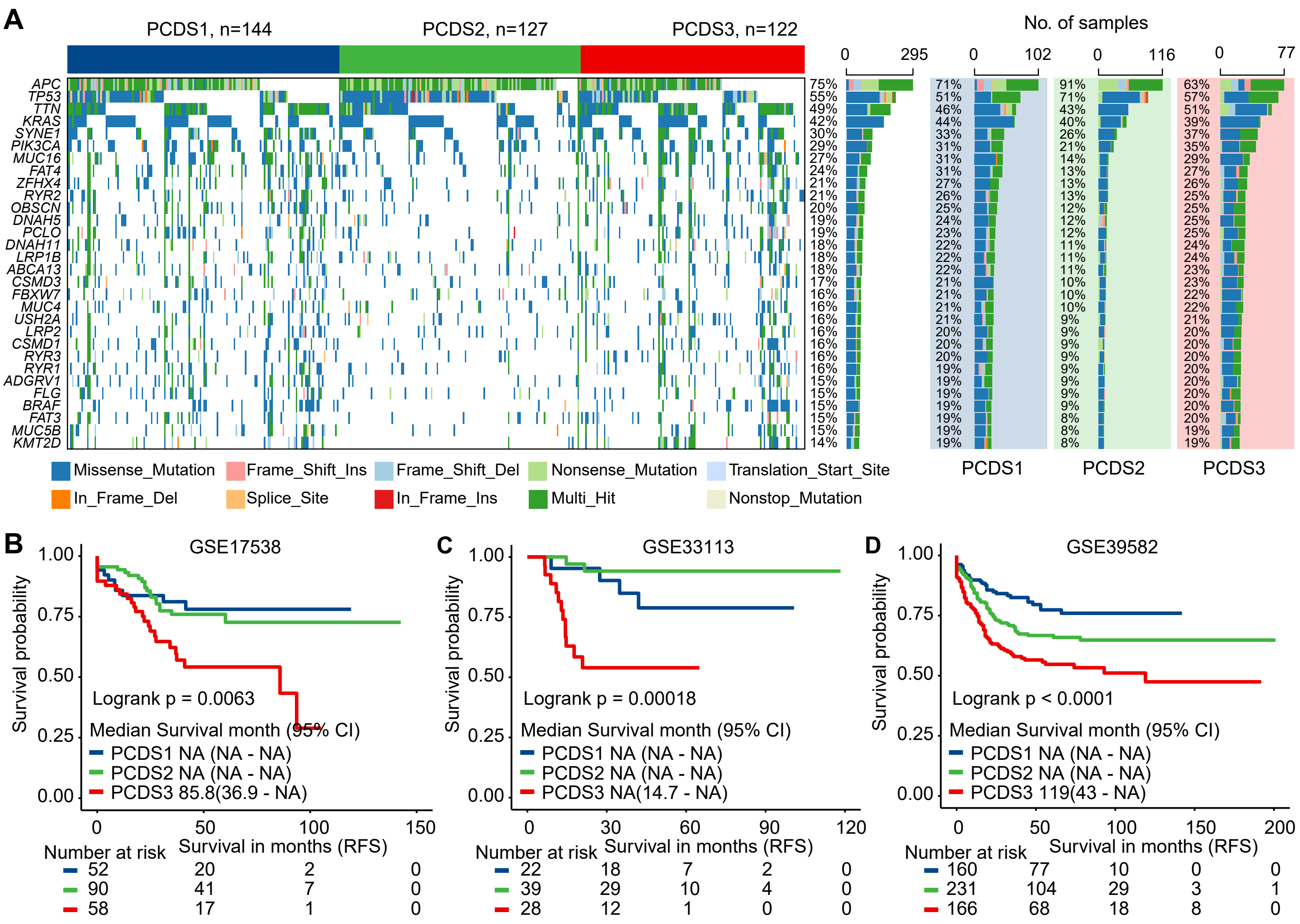

Figure S3. Mutation Landscape in TCGA COAD cohort and Clinical Outcomes of Independent GEO COAD Cohort. (**A**) (left) Oncoplot of top 50 driver gene mutational profiles across three PCDSs from the TCGA COAD cohort. (right) The total mutation frequency of each gene and its mutation frequency in different PCDS patients. (**B-D**) Relapse-free survival (RFS) stratification by PCDS across three independent COAD cohorts, including GSE17538 (B), GSE33113(C) and GSE39582 (D). P values were obtained by the log rank test.

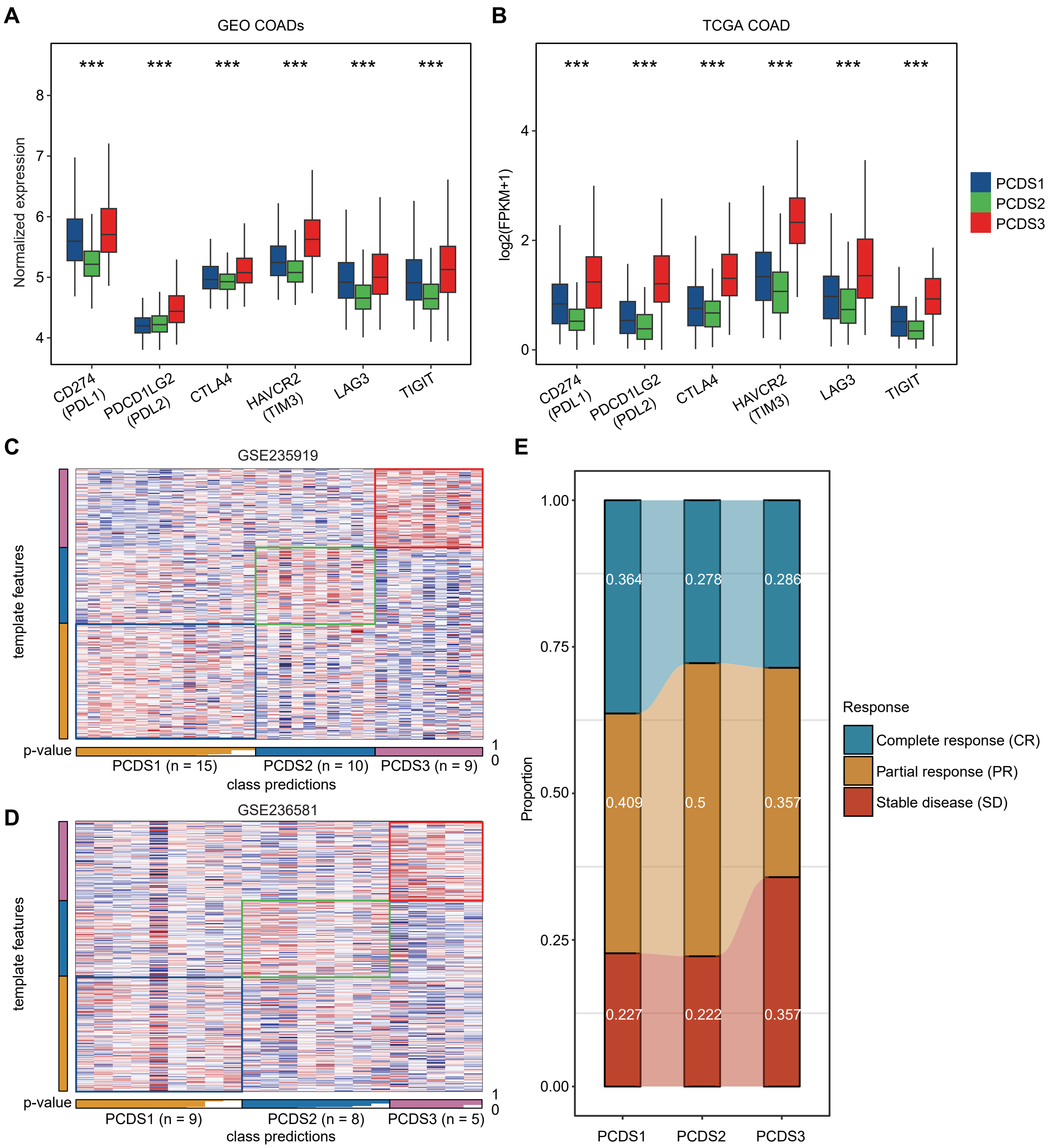

Figure S4. The expression of immune checkpoints and immunotherapy responses to ICB of three PCDSs. (**A, B**) Comparison of the expression of selected immune checkpoint and exhaustion markers across 3 PCDSs in GEO (A) and TCGA (B) COAD cohorts. P values were calculated via one-way analysis of variance. ***, p <= 0.001; **, p <= 0.01; *, p <= 0.05; ·, p <= 0.1; ns, p > 0.1. (**C, D**) Heatmap of the template feature expression level between 3 PCDSs in the GSE235919 and GSE236581 cohorts. (**E**) Stacked bar plot demonstrating proportion of CR, PR, and SD according to three PCDSs from the GSE235919 and GSE236581 cohorts.

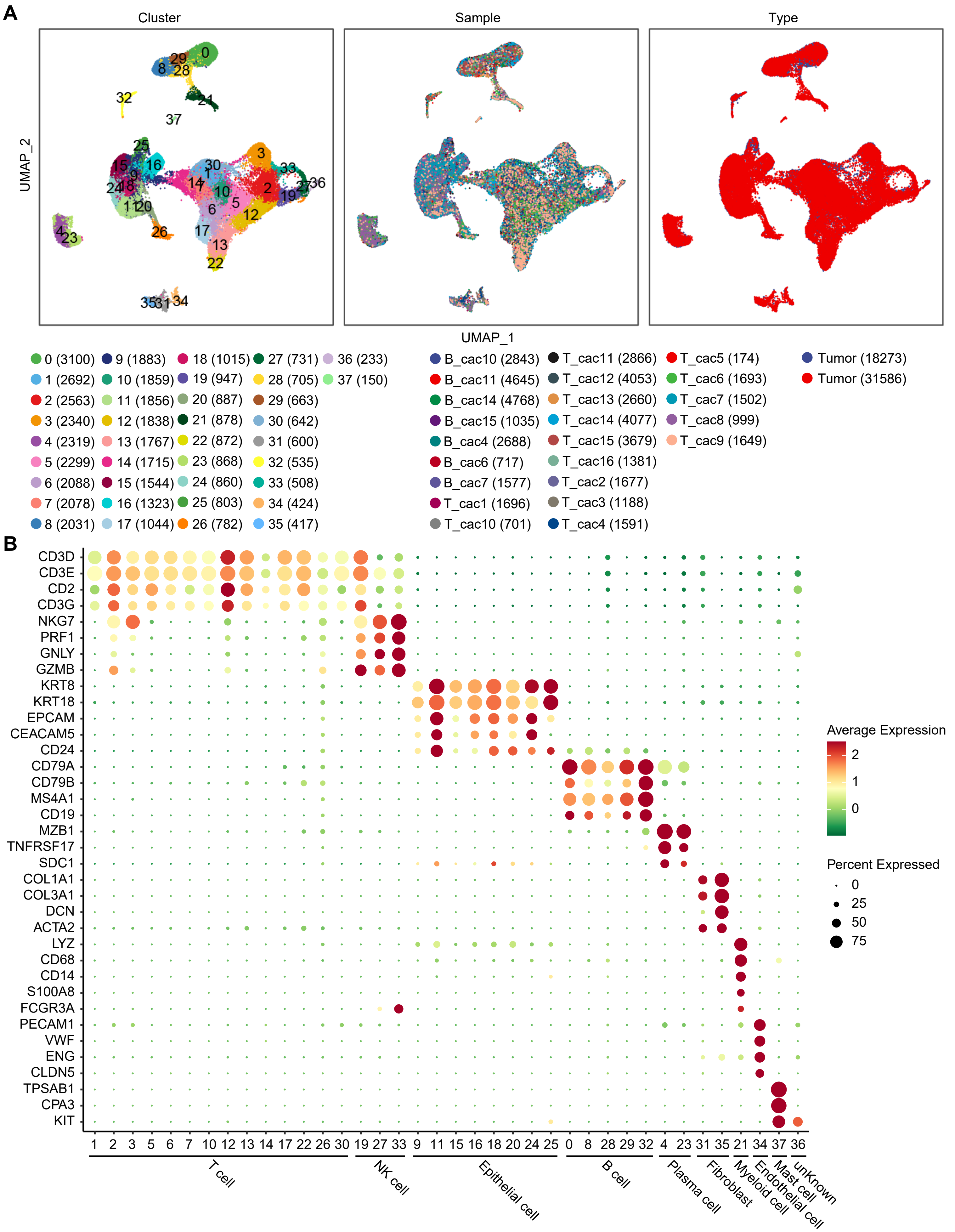

Figure S5. Basic information of 38 major clusters in normal and tumor tissues. (**A**) The UMAP plots showing 38 cell clusters identified by integrated analysis, along with the distribution of cell origins across samples and tissue types. Each dot corresponds to a single cell. (**B**) Dot plots showing average expression of known markers in 38 cell clusters. The dot size represents percent of cells expressing the genes in each cluster. The expression intensity of markers is shown.

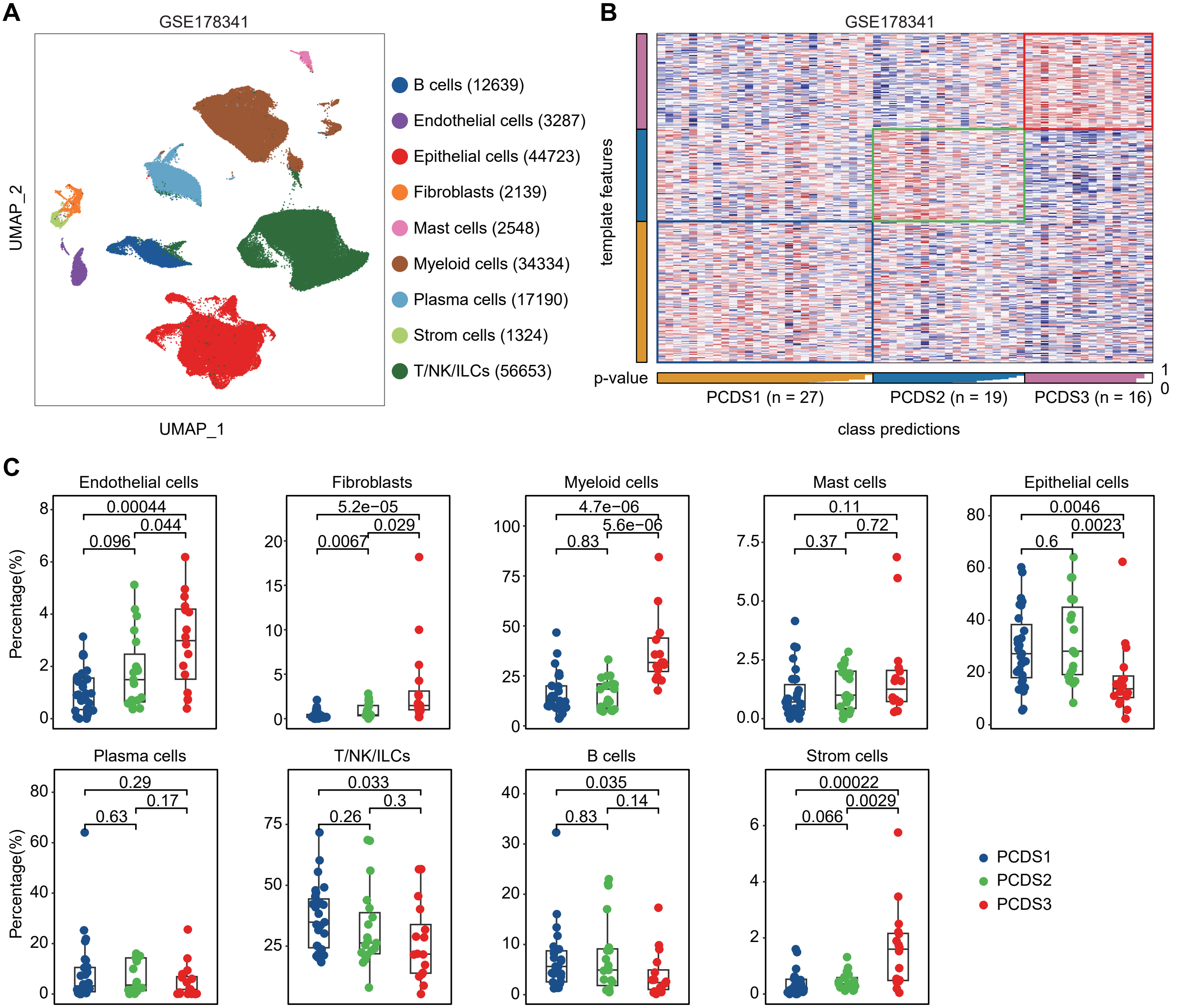

Figure S6. Independent validation of PCDSs classification and associated cellular compositions in the GSE178341 cohort. (**A**) Uniform Manifold Approximation and Projection (UMAP) plot showing major 9 cell types of n = 62 patients identified by original study. Each dot corresponds to a single cell, colored by cell types. (**B**) Heatmap of the template feature expression level between 3 PCDSs in the GSE178341 cohort. (**C**) Comparison of the infiltration abundances of 9 major cell types in three PCDSs. P value was obtained by the Wilcoxon rank-sum test.

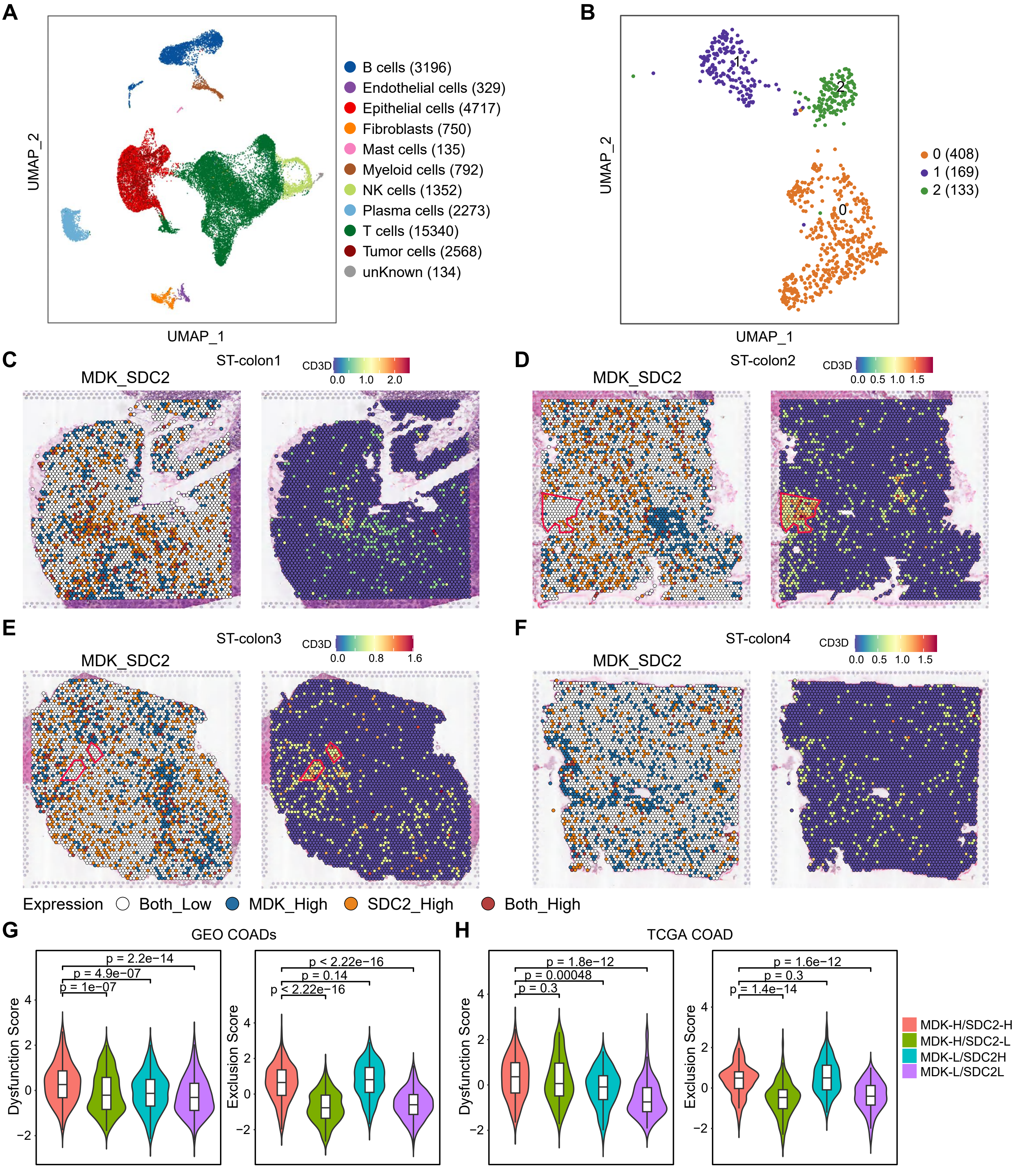

Figure S7. Spatial co-localization of tumor cells and fibroblasts. (**A**) UMAP plot showing the distribution of tumor cells identified by CopyKAT in epithelial cells. Each dot corresponds to a single cell, colored by cell types. NK cells, natural killer cells. (**B**) UMAP plot of individual fibroblasts. Each dot denotes one cell; color represents subcluster. (**C-F**) Visualization of the MDK-SDC2 interaction (left) and the CD3D expression (right) for Spatial transcriptome (ST) data from 4 COAD patients, including ST-colon1 (C), ST-colon2 (D), ST-colon3 (E), and ST-colon4 (F). (**G-H**) Comparison of the T-cell dysfunction and exclusion scores among different MDK/SDC2 expression groups in GEO (G) and TCGA (H) COAD cohorts. P value was obtained by the Wilcoxon rank-sum test.
